# Efficacy and safety of direct-acting antiviral regimen for patients with hepatitis C virus genotype 2: a systematic review and meta-analysis

**DOI:** 10.1186/s12876-024-03414-5

**Published:** 2024-09-30

**Authors:** Pek Kei Lei, Zicheng Liu, Carolina Oi Lam Ung, Hao Hu

**Affiliations:** 1 State Key Laboratory of Quality Research in Chinese Medicine, Institute of Chinese Medical Sciences, University of Macau, Macao SAR, China; 2 Centre for Pharmaceutical Regulatory Sciences, University of Macau, Macao SAR, China; 3 Department of Public Health and Medicinal Administration, Faculty of Health Sciences, University of Macau, Macao SAR, China

**Keywords:** Direct-acting antiviral, Hepatitis C virus, Meta-analysis, Clinical trial

## Abstract

**Background:**

Direct-acting antivirals (DAAs) show high cure rates in treating chronic hepatitis C virus (HCV). However, the effect of DAAs on patients infected with genotype 2 (GT2) is difficult to determine despite the availability of several DAA regimens.

**Methods:**

A systematic search of six databases (PubMed, Embase, Cochrane Library, Web of Science, CNKI, and Clinicaltrial.gov) was conducted through April 20, 2022. We considered the sustained virological response 12 weeks after treatment (SVR12) as the efficacy outcome, and adverse events (AEs) as the safety outcome. By calculating the mean SVR12 and the proportion of AEs among patients, we considered the intervention effect for each DAA regimen. The random effect model was then used in all meta-analyses. This systematic review and meta-analysis aimed to summarize the evidence on efficacy and safety of DAAs in patients infected with HCV GT2. The Bayesian Markov Chain Monte Carlo (MCMC) network metanalysis was used to indirectly compare regimen in GT2 patients.

**Results:**

Among 31 articles included (2,968 participants), consisting of 1,387 treatment-naive patients and 354 patients with cirrhosis. The overall pooled SVR12 rate was 94.62% (95% CI: 92.43-96.52%) among the participants who received all doses of treatment. Meta-analysis results of AEs revealed that fatigue was the most common AE (14.0%, 95% CI: 6.4-21.6%), followed by headache (13.1%, 95% CI: 9.2-17.1%), whereas death and serious adverse events were uncommon.

**Conclusions:**

We compared DAA-based treatments indirectly using meta-analysis and found the combination of Sofosbuvir plus Velpatasvir and Glecaprevir plus Pibrentasvir, each administered over a 12-week period, were identified as the most effective and relatively safe in managing chronic hepatitis C virus genotype 2 (HCV GT2) infection. Both treatments achieved a SVR12 of 100% (95% CI 99–100%).

**Supplementary Information:**

The online version contains supplementary material available at 10.1186/s12876-024-03414-5.

## Background

Hepatitis C virus (HCV) is a blood-borne pathogen that could cause both chronic and acute hepatitis infection; around 50–80% of patients would develop a chronic inflammatory condition, which may lead to liver cirrhosis and hepatocellular carcinoma [1, 2]. Despite the implementation of universal precautions and blood safety measures, HCV infection continues to be a severe global public health burden [3]. The global estimated viraemic prevalence for hepatitis C virus infection was 0.7% (95% UI, 0.7–0.9), corresponding to 56.8 million (95% UI, 55.2–67.8) people infected with HCV in 2020 [4]. Besides, no prophylactic vaccine is available to prevent HCV infection, so the strategy to control HCV has to follow the treatment-as-prevention principle [1].

Since the emergence of well-tolerated oral direct-acting antivirals (DAAs) in early 2014, several major guidelines have recommended DAAs as the first-line treatment for patients infected with HCV instead of pegylated interferon (PEG-IFN) treatment, but therapy options vary according to factors such as HCV genotypes and patients’ status [5–7]. As a member of the *Flaviviridae* family, HCV is heterogeneous and can be classified into 7 genotypes and 67 subtypes, with genotype 1 being the most prevalent in the Americas, Europe, Australia, New Zealand, Central Asia and East Asia [8, 9]. Hepatitis C virus genotype 2 (HCV GT2) is the third predominant genotype in Asia, Africa and America, whereas the prevalence rate varies by geographical distribution, ranging from 62.9% in West Sub-Saharan Africa to 0.8% in North Africa and Middle East [10].

Nevertheless, compared with genotype 1 and 3, the evidence base for DAA therapies in GT2 patients is less extensive. Although there has been clinical evidence to support the novel DAAs entering the market, the majority of trials are open-label, single-arm studies that lack placebo comparators and primarily focus on individual DAA regimens. The comparative efficacy of individual combination therapies remains largely undetermined, primarily due to the scarcity of head-to-head trials Thus far, no studies have compared the efficacy and safety of all DAAs regimens for treating patients infected with HCV GT2.

To fill these gaps, this systematic review and meta-analysis focused on the studies about DAAs treatment for HCV GT2, to assess the comparative efficacy of DAAs regimens, and to identify the benefits and AEs associated with each DAAs intervention.

## Methods

The Preferred Reporting Items for Systematic Reviews and Meta-Analyses Protocols guidelines (PRISMA-P) were adhered to this meta-analysis and systematic review [11]. This systematic review was registered on the PROSPERO database for systematic reviews (CRD: 42022344032).

### Eligibility criteria

We predefined criteria for inclusion in accordance with PICOS principles [12]. Clinical studies which investigated DAAs regimens for treating patients infected with HCV GT2 were eligible for inclusion. The involved population must be adults with diagnosed HCV GT2 infection, who never had HIV/HCV co-infected or decompensated cirrhosis. The detailed eligibility criteria are listed in Table 1.

**Table 1:** Eligibility criteria based on PICO

Characteristics	Eligibility criteria
Population	Aged over 18 years old with HCV GT2 infection.
	Limitation on children/adult patients with HIV/HCV co-infected or patients with decompensated cirrhosis
Interventions	The intervention group included at least one DAA, either as monotherapy or in combination with other treatments.
Comparators	Not applicable
Outcome measures	Primary outcome measures: Sustained virological response 12 weeks after completion of treatment, SVR12
	Secondary outcome measures: All adverse events associated with DAAs treatment (Including non-severe adverse events and serious adverse events)
Study type	Clinical trial
Restrictions	Language of studies in Chinese or English

## Search strategy

Six databases, including PubMed, Embase, Cochrane Library, Web of Science, CNKI, and Clinicaltrial.gov were searched for the relevant clinical trials. The last systematic search was performed on April 20, 2022. Search strategies were constructed on: (i) population: Chronic, Hepatitis C virus; (ii) Interventions: direct-acting antivirals, DAAs; (iii) study design: clinical trial, including single-arm studies (Supplementary Table S1).

### Study selection

The process of selecting studies included two parts, through the literature software EndNote X9. Two reviewers independently screened articles by title and abstract based on eligibility criteria after removing duplicate records. Then, two researchers kept reviewing the remaining literature by full text. Any disputes between the two independent reviewers were resolved through discussion with the senior author.

### Data extraction

Two researchers extracted information from the included studies independently. A third researcher resolved any further disagreements. The data extraction form comprises:

i. study design: author, NCT number, published year and country.
ii. study design: population and design.
iii. characteristics of patients: intervention, duration, with/without cirrhosis, treatment-experienced, treatment-naïve.
iv. outcome measures: SVR12, the number of AEs and SAEs.
v. any information for the assessment of the risk of bias.

### Quality assessment

The RoB 2 tool was used for the methodological quality evaluation of the randomized controlled trials (RCTs), which evaluated the following five domains: the randomization process, deviations from intended interventions, missing outcome data, measurement of the outcome and selection of the reported result [13]. An outcome of low risk, high risk, or some concerns was reported for each domain. An overall assessment of the risk of bias was then determined for each study.

As for non-randomized studies, we used the risk of bias tool for nonrandomized studies (ROBINS-I) proposed by Cochrane Collaboration to assess the following five domains: (i) misclassification of interventions; (ii) deviations from intended intervention; (iii) missing data; (iv) measurement of outcomes; and (v) selection of the reported result [14]. An outcome of low, moderate, serious, critical, or no information for bias risk was reported for each domain. The combination of the five domains was then used to determine the overall risk of bias, whereas judgement for each domain was based on information extracted from each article.

Two researchers assessed the quality of all included studies for the quality assessment process independently. Any disagreements between the two independent reviewers were resolved by discussion and/or consultation with another researcher.

### Statistical analyses

The SVR12 was regarded as the primary efficacy outcome, while the AEs was regarded as the secondary safety outcome. We extracted study-arm data from each study and summarised the intervention effect by calculating the proportion of patients reaching the SVR12 or with any AEs over the number of involved patients. Firstly, the overall pooled arm-specific proportion was conducted regardless of the cirrhosis status of patients, previous history of treatment, or treatment type. Then, subgroup analyses by all the above variables were conducted for specific efficacy outcomes. As for safety outcomes, any AEs reported were recorded and considered. Meta-analyses for SVR12 were conducted by using Freeman-Tukey double arcsine transformation, and a random effect model was performed for all meta-analyses because SVR12 rates were expected to follow the binomial distribution and approach the extreme boundaries [15]. The Bayesian Markov Chain Carlo (MCMC) was used to conduct the network meta-analysis. The MCMC approach was utilized three chains and was refined through 200,000 simulations, with every tenth simulation retained and the first 10,000 discarded as burn-in. We used software R v.4.1.2 and package ‘Meta’ v.5.2-0 & ‘gemtc’ to perform the meta-analysis, estimating and pooling the rate of SVR12 and AEs [16]. Unless otherwise stated, we set two-tailed statistical significance as *P* values < 0.05 for all analyses.

### Assessment of heterogeneity

Heterogeneity of treatment effects was quantified by *I*^*2*^ statistics, with thresholds of 25%, 50% and 75%, where heterogeneity suggests low if (25%≤ *I*^*2*^ < 50%), moderate (50%≤ *I*^*2*^ < 75%), or high (*I*^*2*^ ≥ 75%), respectively [17].

## Results

### Characteristics of included studies

A total of 4,486 studies were yielded from database search, of which 703 duplicates were removed. After applying our inclusion criteria, we identified 31 articles for our meta-analysis, which included 35 clinical studies. All included articles were published within the past 10 years, with the majority being recent studies published within the past 5 years.

**Fig. 1:**
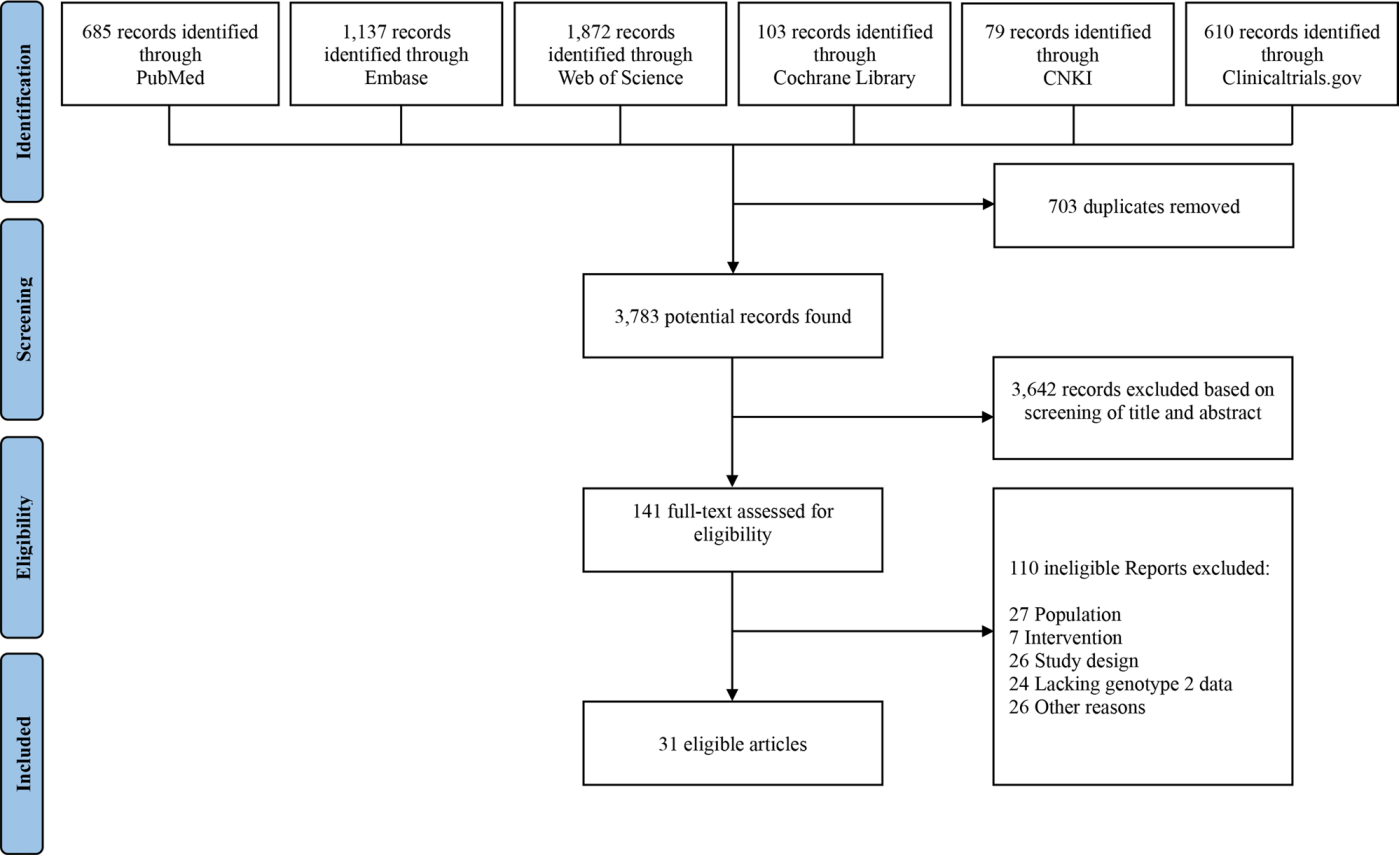
Flowchart summarizing the selection and identification of studies based on PRISMA

In accordance with the Preferred Reporting Items for Systematic Reviews and Meta-Analyses (PRISMA) guidelines, our search strategy and selection process are depicted in Fig. 1. Our search yielded a total of 31 articles, consisting of 9 randomized controlled trials (RCTs) and 26 non-randomized studies. Of the 35 clinical trials, 13 were conducted in phase 2, while 21 were conducted in phase 3; 1 clinical trial was conducted in phase 4. All 35 included studies were multicenter clinical trials.

2,968 participants with HCV GT2 from these 35 studies were eligible for the data synthesis, as presented in Table 2, consisting of 1,389 treatment-naive patients and 354 patients with cirrhosis. The sample size of the included clinical trials ranged from 18 to 458. All participants were diagnosed with HCV GT2 infection and received DAA therapy as their primary treatment.

**Table 2:** Characteristics of included studies for systematic review and meta-analysis

Author	NCT Number	Year	Country	Population and design	Intervention	Participants (*n*)	Cirrhosis	Duration	SVR12	Sponsors and Collaborators
							*n* (%)	(week)	*n* (%)	
Lawtiz et al., 2015 [48]	NCT01458535	2015	NA	Non-Randomized, open-label, sequential arm, phase 2, multicenter study, TN G1-3 Patients	OBV+PTV+RTN±RBV	20	0	12	14(70.0%)	AbbVie
Jacobson et al., 2013 [24]	NCT01542788 (POSITRON)	2013	NA, ANZ	Randomized, blinded, placebo-controlled, phase 3, multicenter study, TN G2-3 patients	SOF+RBV	143	17(11.9%)	12	101(92.7%)	Gilead Sciences
Jacobson et al., 2013 [24]	NCT01604850 (FUSION)	2013	NA, ANZ	Randomized, open-label, active control, phase 3, multicenter study, TE G2-3 patients	SOF+RBV	68	19(27.9%)	12 or 16	61(89.7%)	Gilead Sciences
Lawitz et al., 2013 [25]	NCT01497366(FISSION)	2013	NA, EU, ANZ	Randomized, Open-label, multicenter, Phase 3 RCT, TN G1-3 Patients	SOF+RBV/PR	137	N/A	12	120(87.6%)	Gilead Sciences
Zeuzem et al., 2014 [27]	NCT01682720 (VALENCE)	2014	EU	Randomized, blinded, placebo-controlled, phase 3, multicenter, TN/TE G2-3 patients	SOF+RBV	91	11(12.1%)	12	68(93.2%)	Gilead Sciences
Foster et al., 2016 [49]	NCT01616524 (PRINCIPAL)	2016	NA, LM, ASIA, EU	Randomized, blinded, controlled, phase 3, multinational, TN G2-3 patients	Lambda+RBV+DCV	458	29(6.3%)	12 or 24	363(79.3%)	Bristol-Myers Squibb
Omata et al., 2014 [26]	NCT01910636	2014	Japan	Open-label, phase 3, multicenter, TN/TE G2 patients	SOF+RBV	153	17(11.1%)	12	148(96.7%)	Gilead Sciences
Brown et al., 2017 [50]	NCT01932762(C-SCAPE)	2017	N/A	Randomized, open-label, phase 2, multi-site, TN G2,4,5,6 patients	EBR±GZR±RBV	56	0	12	43(76.8%)	Merck Sharp & Dohme LLC
Dore et al., 2015 [51]	NCT01257204(AI444-031)	2015	NA, EU, ANZ	Randomized, blinded, placebo-controlled, phase 2, multicenter, TN G2-3 patients	DCV+PEG+RBV	71	68(95.8)	12 or 16 or 24	57(80.3%)	Bristol-Myers Squibb
Everson et al., 2015 [52]	NCT01858766	2015	NA	Randomized, open-label, phase 2, multicenter, TN G1-6 patients	SOF+VEL	103	0	8	88(65.2%)	Gilead Sciences
Feld et al., 2015 [19]	NCT02201940(ASTRAL-1)	2015	NA, EU, ASIA	Randomized, blinded, placebo-controlled, phase 3, multicenter, TN/TE G1-2, 4-5 patients	SOF+VEL	125	N/A	12	104(100%)	Gilead Sciences
Foster et al., 2015 [20]	NCT02220998(ASTRAL-2)	2015	NA	Randomized, open-label, phase 3, multicenter, TN/TE G2 patients	SOF+VEL/SOF+RBV	266	38(14.3%)	12	257(96.6%)	Gilead Sciences
Foster et al., 2015 [53]	NCT01962441	2015	NA, EU, ANZ	Randomized, open-label, phase 3, multicenter, TN/TE G2-3 patients	SOF+RBV+PEG	48	N/A	12 or 16 or 24	45(93.8%)	Gilead Sciences
Asselah et al., 2018 [32]	NCT02243293(SURVEYOR-II)	2018	N/A	Randomized, open-label, phase 3, multicenter, TN/TE G2 patients	GLE+PIB	145	0	8	142(97.9%)	AbbVie
Asselah et al., 2018 [32]	NCT02640482(ENDURANCE-2)	2018	N/A	Randomized, blinded, placebo-controlled, phase 2, multicenter, TN/TE G2 patients	GLE+PIB	302	0	12	201(99.5)	AbbVie
Gane et al., 2016 [54]	NCT02378961	2016	NA, ANZ	Non-Randomized, open-label, phase 2, multicenter, TN/TE G2-3, 6 patients	SOF+VEL+VOX	33	14(42.4%)	6 or 8 or 12	31(93.9%)	Gilead Sciences
Lawitz et al., 2017 [55]	NCT02332707	2017	N/A	Randomized, open-label, phase 2, multicenter, TN G1-3 patients	GZR+RZR+UPR±RBV	151	57(37.7%)	8 or 12	141(93.4%)	Merck Sharp & Dohme Corp
Toyoda et al., 2018 [30]	NCT02707952(CERTAIN-1)	2018	ASIA	Randomized, open-label, phase 3, multicenter, TN/TE G2 patients	GLE+PIB	18	18(100%)	12	18(100%)	AbbVie
Toyoda et al., 2018 [30]	NCT02723084(CERTAIN-2)	2018	ASIA	Randomized, open-Label, active comparator, phase 3 multicenter, TN/TE G2 patients	GLE+PIB/SOF+RBV	136	0	8	88(97.8%)	AbbVie
Gane et al., 2017 [31]	NCT02202980(LEPTON)	2017	ANZ	Non-randomized, open-label, phase 2, multicenter, TN/TE G2 patients	LDV+SOF	53	2(3.8%)	8 or 12	45(84.9%)	Gilead Sciences
Ho et al., 2017 [28]	NCT02128542(VALOR-HCV)	2017	NA	Open-label, phase 4, multicenter, TN/TE G2 patients	SOF+RBV	66	66(100%)	12	52(78.9%)	Gilead Sciences
Jacobson et al., 2017 [21]	NCT02607800(POLARIS-2)	2017	NA, ANZ, EU	Randomized, open-label, phase 3, multicenter, TN G1-6 patients	SOF+VEL+VOX/SOF+VEL	116	N/A	8 or 12	114(98.3%)	Gilead Sciences
Lawitz et al., 2019 [56]	NCT02956629(MK-3682-041)	2019	N/A	Non-randomized, open-label, phase 2, multi-site, TN/TE G1-6 patients	RZR+UPR	47	10(21.3%)	12	43(91.5%)	Merck Sharp & Dohme Corp
Lawitz et al., 2019 [57]	NCT02759315(MK-3682-035)	2019	NA	Non-randomized, open-label, phase 2, multi-center TN/TE G1-6 patients	RZR+UPR	29	7(24.1%)	12	28(96.6%)	Merck Sharp & Dohme LLC
Wei et al., 2018 [58]	NCT02671500	2018	ASIA	Open-label, Phase 3, multicenter, TN & TE G1-6 Patients	SOF+VEL	64	N/A	12	64(100%)	Gilead Sciences
Wei et al.,2020 [34]	NCT03222583(VOYAGE-1)	2020	ASIA	Randomized, blind, placebo-controlled, phase 3, multicenter, TN/TE G1-6 patients	GLE+PIB	210	0	8	136(97.8%)	AbbVie
Wei et al.,2020 [34]	NCT03235349(VOYAGE-2)	2020	ASIA	Open-label, multicenter, phase 3, TN/TE G1-6 patients	GLE+PIB	53	53	12	53(100%)	AbbVie
Asahina et al., 2018 [29]	N/A	2018	ASIA	Randomized, open-label, phase 3, multicenter, TN & TE G2 patients	LDV+SOF/SOF+RBV	131	18(13.7%)	12	126(96.2%)	Gilead Sciences
Izumi et al., 2018 [59]	N/A	2018	ASIA	Open-label study, phase 3, multicenter, TE G1-2 patients	SOF+VEL+RBV	22	5(22.7%)	12 or 24	18(81.8%)	Gilead Sciences
Rao et al., 2019 [60]	NCT03416491(KW-136_II)	2019	ASIA	Randomized, open-label, phase 2, multicenter, TN G1-3,6 patients	CLV+SOF	27	4(14.8%)	12	26(96.3%)	Beijing Kawin Technology
Shafran et al., 2017 [61]	NCT02292719(QUARTZ II-III)	2017	N/A	Randomized, open-label, phase 2, multicenter, TN/TE G2-3 patients	OBV+PTV+RTN+SOF±RBV	19	0	6 or 8	13(68.4)	AbbVie
Brown et al., 2020 [33]	NCT03089944(EXPEDITION-8)	2020	NA, EU, ASIA	Open-label, phase 3b, multicenter, TN G1-6 Patients	GLE+PIB	26	N/A	8	26(100%)	AbbVie
Asselah et al.,2019 [22]	NCT02346721	2019	NA, EU, ASIA	Open-label, phase 3, multicenter, TN & TE G1,2,4,6 Patients	SOF+VEL	20	N/A	12	20 (100%)	Gilead Sciences
Gao et al.,2020 [62]	NCT03995485(KW-136_III)	2020	ASIA	Open-label, phase 3, multicenter, TN & TE G1-3, 6 Patients	CLV+SOF	95	N/A	12	91(95.8%)	Beijing Kawin Technology
Hua et al., 2022 [63]	NCT04070235	2022	ASIA	Randomized, open-label, phase 2, multicenter, TN & TE G1-3, 6 Patients	AFV+DCV	31	N/A	12	31(100%)	Nanjing Sanhome Pharmaceutical

AFV: Alfosbuvir; SOF: Sofosbuvir; VEL: Velpatasvir; LDV: Ledipasvir; CLV: Coblopasvir; DCV: Daclatasvir; GLE: Glecaprevir; PIB: Pibrentasvir; EBR: Elbasvir; GRZ: Grazoprevir; RUZ: Ruzasvir; RTN: Ritonavir; UPR: Uprifosbuvir; VOX: Voxilaprevir; PR: Peginterferon and Ribavirin; RBV: Ribavirin; NA: North America; EU: Europe; ASIA: Asia; ANZ: Australia and New- Zealand; AFR: Africa, LM: Latin Ameica; TN: Treatment-naïve; TE: Treatment-experienced; NIH: National Institutes of Health; G: Genotype; N/A: Not applicable; RCT: Randomized controlled trials; NCT: ClinicalTrials.gov Identifier;

A total of 23 combinations of DAAs were investigated, duration of therapy ranged from 6 to 16 weeks with or without the addition of ribavirin. We excluded the 6-week regimens from our meta-analysis due to a limitation on sample size (*n* = 6). Among the 35 clinical trials included in this study, DAAs were regarded as the first-line treatment. According to the mechanism of action, DAAs were divided into four types [18]:

i. NS5A Inhibitor (Daclatasvir, Elbasvir, Ledipasvir, Ombitasvir, Ledipasvir, Pribrentasvir, Velpatasvir).
ii. Protease NS3/4A Inhibitor (Glecaprevir, Paritaprevir, Simeprevir, Grazoprevir, Voxilaprevir).
iii. Non-Nucleoside NS5B Polymerase Inhibitor (Dasabuvir, Deleobuvir).
iv. NS5B Polymerase Inhibitor (Sofosbuvir).

### Quality assessment

There are two domains were considered at low risk of bias in all studies, including classification of intervention and measurement of outcome (Supplementary Table S2 & Table S3). Of the 26 non-randomized trials included in our analysis, the quality was evaluated using the ROBINS-I tool, which assesses bias from five perspectives. Among these 26 studies, 12 were judged to be at moderate risk of bias due to deviations from the intended interventions. Bias due to missing data was considered moderate in 10 studies. Of the 9 randomized controlled trials assessed using the ROB 2 tool, 2 were judged to be at high risk of bias due to selection and performance. Overall, only 1 out of the 9 RCTs was considered to be at high risk of bias (Supplementary Figure S1).

### Overall pooled SVR12 for DAA therapies

**Fig. 2:**
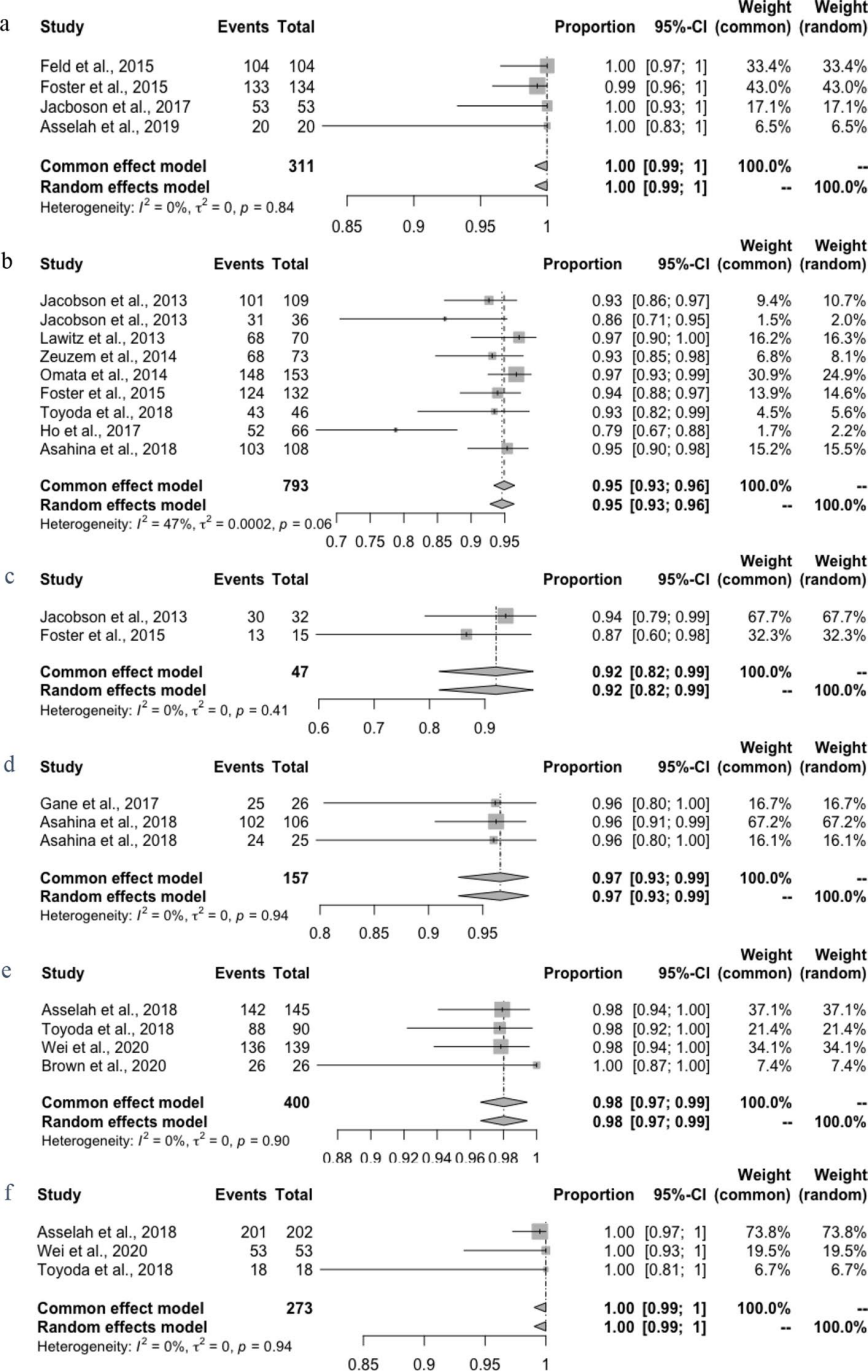
Forest plot of proportions of HCV GT2 patients reaching SVR12 weeks after the end of treatment with DAAs over patients receiving all doses of treatment, according to type of treatment. DAA regimen of SOF + VEL-12(**a**), SOF + RBV-12(**b**), SOF + RBV-16(**c**) LDV + SOF-12(**d**), GLE + PIB-8(**e**) and GLE + PIB-12(**f**)

Since 2014, multiple regimens have been utilized for the treatment of hepatitis C virus genotype 2, with overall response rates showing considerable success. The characteristics of the 35 included studies (58 study-arms, *n* = 2,968) are reported (Supplementary Table S4). A total of 2,968 GT2 patients who received DAA therapy were included. As seen in Supplementary Figure S2, the pooled SVR12 was 94.62% (95% CI: 92.43-96.51%), but with substantial heterogeneity (*I*^*2*^ = 72.0%, *P* < 0.0001) caused by subgroups. Our network connected 10 mainstream treatment regimens for further indirectly comparison. Figure 2 illustrates the network of studies included in our network meta-analysis, comparing five different regimens. Notably, all regimens were compared directly to at least one other regimen, forming a well-connected network. The connection between SOF + RBV 12 and GLE + PIB 12 is depicted with a moderately thick line, indicating several studies have directly compared these two regimens. In contrast, the thinner line between SOF + VEL + VOX 8 and GLE + PIB 12 suggests fewer direct comparisons, relying more on indirect evidence. The assessment of inconsistency showed no significant differences between direct and indirect comparisons within this network. By comparing the main treatment regimens, we have confirmed that the 12-week SOF + VEL therapy and the 12-week GLE + PIB therapy are essentially equivalent in efficacy and are the treatment regimens with the highest response rates (Supplementary Figure S3).

We also did further analysis to indirectly compare the results between every two regimens (Table 3). Each cell in Table 3 shows the estimated difference in response rates between regimens with the corresponding 95% confidence intervals. For instance, the comparison between SOF + VEL-12 and GLE + PIB-12 shows a value of -1.05 (95% CI: -7.80, 5.58), suggesting that there is no significant difference in response rates between these regimens. We also highlighted interesting findings such as the comparison between SOF + VEL-12 and SOF + VEL + VOX-12, noting that adding voxilaprevir does not significantly change the response rate but may affect other clinical considerations like the duration of treatment or side-effect profile.

**Table 3:** Results of network meta-analysis between regimens in HCV genotype 2 patients (95% CI)

	LDV+SOF-12	LDV+SOF-8	GLE+PIB-12	GLE+PIB-8	SOF+RBV-12	SOF+RBV-16	SOF+RBV-24	SOF+VEL-12	SOF+VEL+VOX-12	SOF+VEL+VOX-8
LDV+SOF-12	**LDV+SOF-12**	-2.54 (-6.95, 1.12)	3.67 (-1.44, 10.44)	1.09 (-3.75, 6.23)	-0.23 (-3.65, 3.19)	-0.06 (-4.31, 4.20)	27.73 (1.04, 94.57)	2.60 (-2.20, 8.12)	-35.13 (-123.10, 29.89)	-18.58 (-65.60, 2.45)
LDV+SOF-8	2.54 (-1.12, 6.95)	**LDV+SOF-8**	6.33 (0.02, 14.32)	3.72 (-2.39, 10.30)	2.35 (-2.68, 7.83)	2.54 (-3.15, 8.50)	30.34 (3.32, 97.32)	5.27 (-0.87, 12.10)	-32.36 (-120.76, 32.76)	-16.02 (-63.14, 5.70)
GLE+PIB-12	-3.67 (-10.44, 1.44)	-6.33 (-14.32, -0.02)	**GLE+PIB-12**	-2.53 (-6.95, 0.17)	-3.88 (-9.65, 0.10)	-3.72 (-10.08, 0.90)	23.82 (-3.30, 90.57)	-1.05 (-7.80, 4.78)	-39.01 (-127.03, 25.80)	-22.63 (-69.96, -0.89)
GLE+PIB-8	-1.09 (-6.23, 3.75)	-3.72 (-10.30, 2.39)	2.53 (-0.17, 6.95)	**GLE+PIB-8**	-1.31 (-5.08, 2.13)	-1.16 (-5.69, 3.07)	26.48 (-0.14, 93.36)	1.54 (-3.57, 7.01)	-36.19 (-124.13, 28.74)	-19.78 (-66.87, 1.46)
SOF+RBV-12	0.23 (-3.19, 3.65)	-2.35 (-7.83, 2.68)	3.88 (-0.10, 9.65)	1.31 (-2.13, 5.08)	**SOF+RBV-12**	0.15 (-2.37, 2.72)	27.90 (1.59, 94.76)	2.80 (-0.64, 7.17)	-34.88 (-122.79, 29.93)	-18.36 (-65.25, 2.35)
SOF+RBV-16	0.06 (-4.20, 4.31)	-2.54 (-8.50, 3.15)	3.72 (-0.90, 10.08)	1.16 (-3.07, 5.69)	-0.15 (-2.72, 2.37)	**SOF+RBV-16**	27.74 (1.36, 94.69)	2.66 (-0.99, 7.14)	-34.92 (-123.25, 29.90)	-18.49 (-65.50, 2.24)
SOF+RBV-24	-27.73 (-94.57, -1.04)	-30.34 (-97.32, -3.32)	-23.82 (-90.57, 3.30)	-26.48 (-93.36, 0.14)	-27.90 (-94.76, -1.59)	-27.74 (-94.69, -1.36)	**SOF+RBV-24**	-24.93 (-92.00, 1.61)	-68.75 (-167.88, 9.81)	-52.21 (-125.13, -7.81)
SOF+VEL-12	-2.60 (-8.12, 2.20)	-5.27 (-12.10, 0.87)	1.05 (-4.78, 7.80)	-1.54 (-7.01, 3.57)	-2.80 (-7.17, 0.64)	-2.66 (-7.14, 0.99)	24.93 (-1.61, 92.00)	**SOF+VEL-12**	-37.77 (-125.59, 26.95)	-21.28 (-68.14, -1.15)
SOF+VEL+VOX-12	35.13 (-29.89, 123.10)	32.36 (-32.76, 120.76)	39.01 (-25.80, 127.03)	36.19 (-28.74, 124.13)	34.88 (-29.93, 122.79)	34.92 (-29.90, 123.25)	68.75 (-9.81, 167.88)	37.77 (-26.95, 125.59)	**SOF+VEL+VOX-12**	12.95 (-44.16, 89.91)
SOF+VEL+VOX-8	18.58 (-2.45, 65.60)	16.02 (-5.70, 63.14)	22.63 (0.89, 69.96)	19.78 (-1.46, 66.87)	18.36 (-2.35, 65.25)	18.49 (-2.24, 65.50)	52.21 (7.81, 125.13)	21.28 (1.15, 68.14)	-12.95 (-89.91, 44.16)	**SOF+VEL+VOX-8**

### SVR12 analysis by participants’ treatment history

**Fig. 3:**
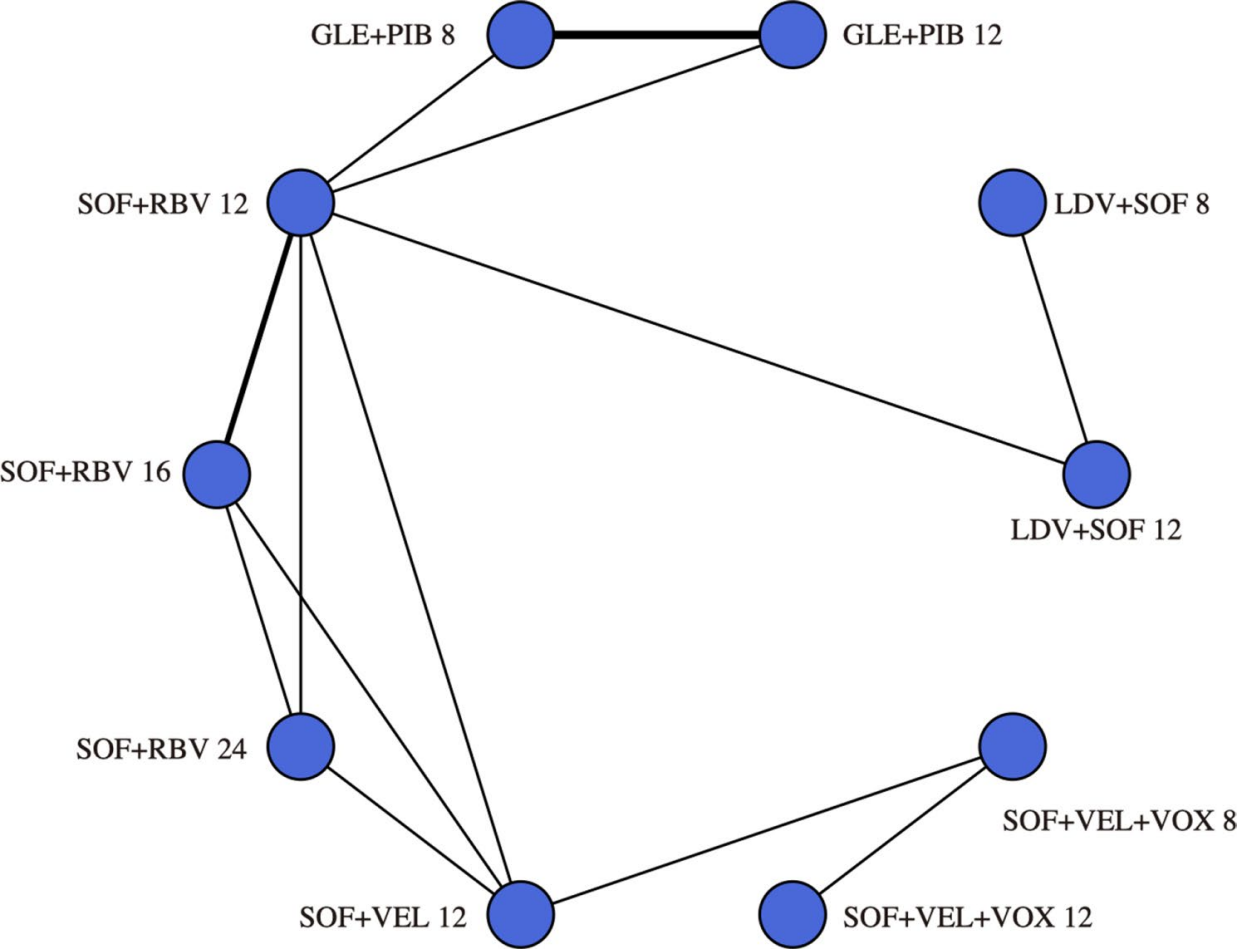
Networks of studies. Evidence network of DAA-based regimens studied in chronic hepatitis C genotype 2 patients (The nodes represent each regimen, with the thickness of the connecting lines represents the number of studies. The edges between nodes indicate direct comparisons, with thicker edges denoting multiple study comparisons)

Totally, 48 study-arms provided data on whether patients had been treated or not, including 1,387 treatment-naïve patients and 414 treatment-experienced patients. As shown in Fig. 3, the pooled SVR12 estimated were 93.18% (95% CI 89.88–95.95%) for the treatment-naïve group and 95.08% (95% CI 89.54–98.94%) for the treatment-experienced group. Both groups show substantial and moderate heterogeneity in *I*^*2*^ = 70% and *I*^*2*^ = 62%, respectively. Compared with treatment-naive patients, treatment-experienced patients had a narrowly 1.9% higher chance of achieving SVR12.

### SVR12 analysis by participants’ cirrhosis status

**Fig. 4:**
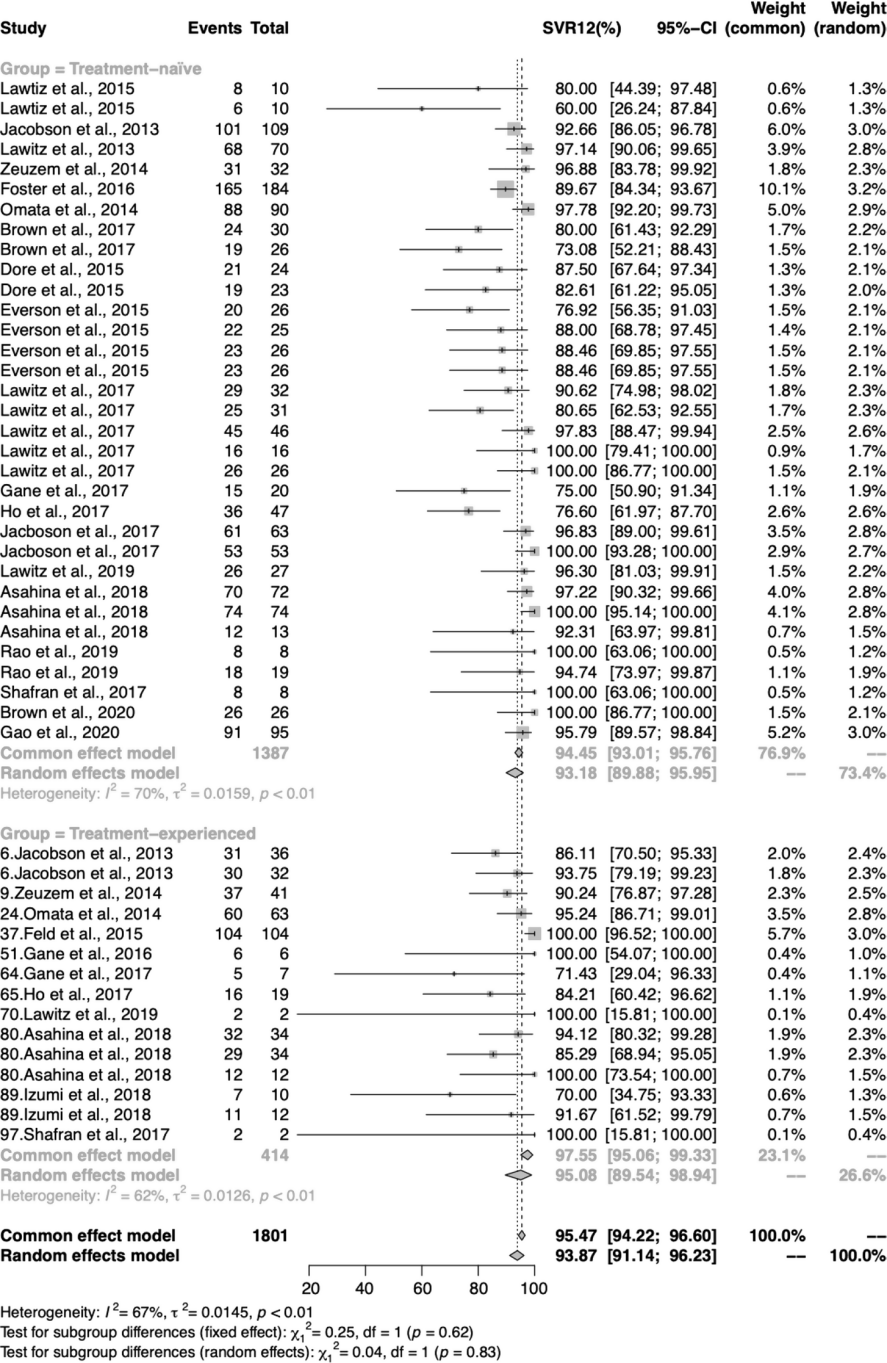
Forest plot of proportions of HCV GT2 patients reaching SVR12 with DAAs, according to pervious history of treatment

Due to the indistinctive difference in treatment history, we moved to focus on the subgroup analysis by cirrhosis status for 381 patients with cirrhosis and 1,709 patients without cirrhosis. In total, 55 study-arms provided data for 20 different regimens in the cirrhotic status subgroup analysis (Fig. 4). Out of expectations, we found that cirrhotic patients had a comparatively high SVR12 of 97.17% (95% CI 93.01%, 99.73%), while non-cirrhotic patients only had 92.77% (95% CI 89.48%, 95.57%).

### Analysis of adverse events for DAA therapies

Meta-analysis of 1198 DAA-treated patients’ AEs showed that fatigue was the most common adverse event (14.0%, 95% CI: 6.4-21.6%) followed by headache (13.1%, 95% CI: 9.2-17.1%), while death and serious AEs were uncommon (Table 4). Most AEs related to DAA regimens were transient, and specific medical intervention was unnecessary.

**Table 4:** Meta-analysis of proportions of all adverse events for HCV genotype 2 patients treated with DAAs regimens

Common AEs	DAA regimen and No. of event/No. of participants									Proportion (95% CI) I^2^ statistic (*P* value)
	OBV+PTV+RTN+SOF+RBV-8 (*n*=1)	OBV+PTV+RTN+SOF+RBV-6 (*n*=1)	EBR/GZR+RBV-12 (*n*=1)	SOF+ VEL-12 (*n*=1)	LDV+ SOF-8 (*n*=1)	LDV+ SOF-12 (*n*=3)	SOF+ RBV-12 (*n*=5)	GLE+ PIB-8 (*n*=2)	GLE+ PIB-12 (*n*=2)	
Fatigue	3/10	6/9	12/30	20/134	5/27	6/157	69/425	17/235	23/220	14.0% [6.4; 21.6] 92.8% (*P*<0.0001)
Headache	6/10	3/9	6/30	24/134	8/27	18/157	65/425	30/235	24/220	13.1% [9.2; 17.1] 76.7% (*P*<0.0001)
Nausea	3/10		5/30	14/134	5/27	7/157	31/425	21/235	15/220	5.7% [2.2; 10.8] 89.6% (*P*<0.0001)
Insomnia	2/10			6/134			22/425			0.2% [0.0; 0.6] 52.0% (*P*<0.001)
Irritability				4/134			9/425			0.1% [0.0; 0.5] 0% (*P* =0.64)
Pruritus		2/9		6/134			18/425	3/235	4/220	1.3% [0.2; 2.4] 53.3% (*P*<0.01)
Nasopharyngitis				8/134		23/157	76/425	9/235	2/220	7.4% [2.5; 12.4] 89.3% (*P*<0.001)
Anaemia			5/30				74/425		1/220	5.7% [1.2; 10.3] (*P*<0.0001)
Blood bilirubin increased							7/425	1/235	2/220	2% [0; 0.8] 58.3% (*P*<0.01)
SAE	1/10	0	1/30	2/134	2/27	4/157	13/425	3/235	3/220	1.5% [0.8; 2.1] 0% (*P* =0.64)
Death	0	0	0	2	0	1	0	0	0	0% [0; 0.2] 0 (*P* =0.87)
Any AE	10/10	9/9	26/30	92/134	22/27	95/157	271/425	133/235	143/220	73.1% [66.6; 79.1] 77.4% (*P*<0.0001)

### Sofosbuvir plus Velpatasvir with a duration of 12 weeks

**Fig. 5:**
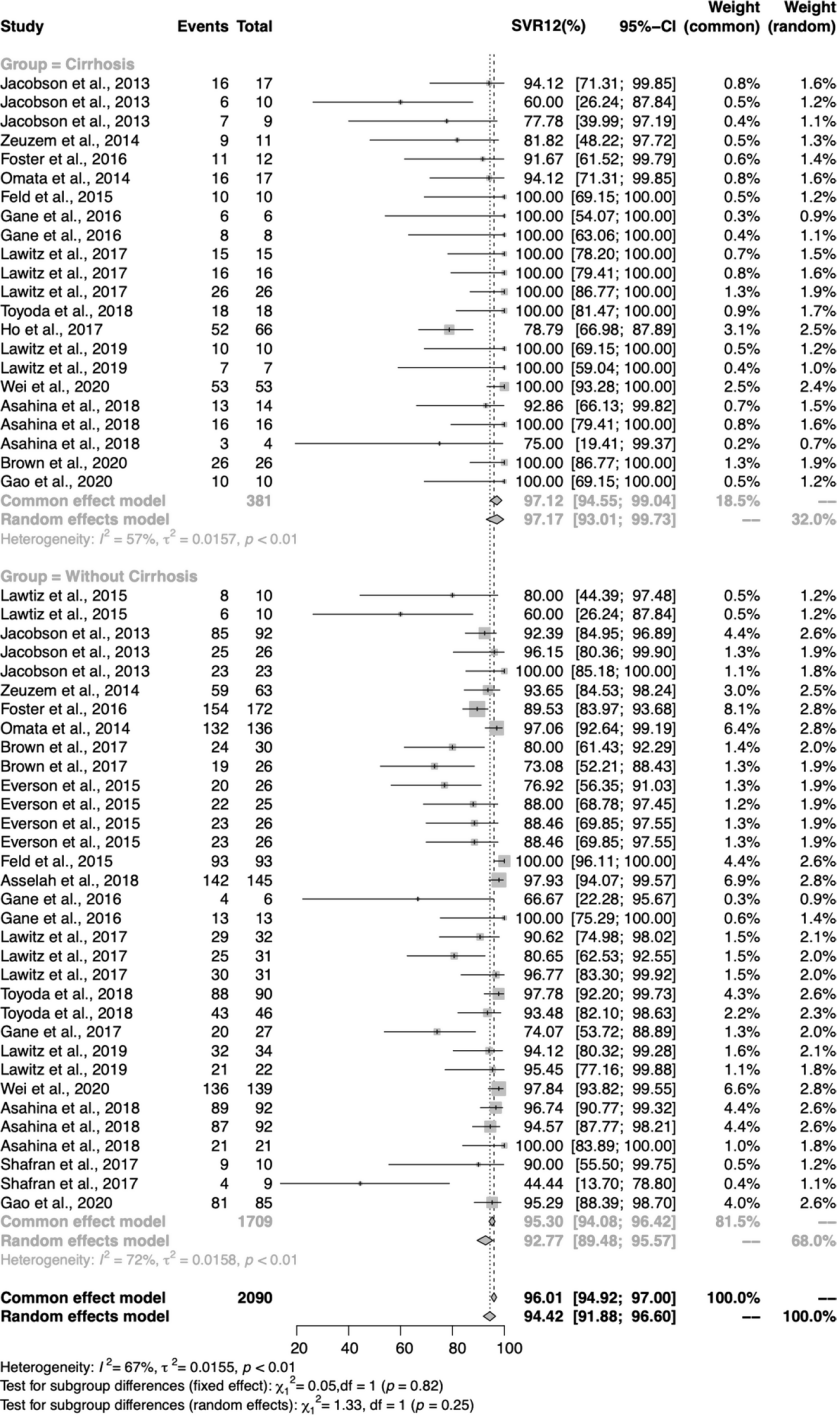
Forest plot of proportions of HCV GT2 patients reaching SVR12 with DAAs, according to cirrhotic status

The highest SVR12 rate was estimated for SOF + VEL for 12 weeks, with SVR12 100% (95% CI 99–100%; Fig. 5a). Among the 311 HCV GT2 patients who treated with SOF + VEL included from 4 arms, any virologic failure was not observed, even in the population with cirrhosis and previous treatment failure [19–22]. Only one study-arm reported the safety profile of SOF + VEL with 9 common AEs being headache (17.9%), fatigue (14.9%), nasopharyngitis (6.0%), nausea (10.4%), pruritus (4.5%), insomnia (4.5%), irritability (3.0%), cough (3.0%), and dyspepsia (0.7%) [23]. The rate of AEs is 68.7%, which was slightly lower among patients treated with SOF + VEL than patients’ pooled level (Supplementary Table S5). However, it should be noted that although 2 out of 134 participants were reported to have died during the post-treatment follow-up, further investigation revealed that the cause of death was attributed to metastatic cancer and cardiac arrest rather than the SOF + VEL treatment itself [20].

### Sofosbuvir plus Ribavirin with a duration of 12 and 16 weeks

As for the SOF + RBV regimen, the treatment of SOF + RBV for 12 weeks was used in 793 patients with HCV GT2 from 9 study-arms, which resulted in a pooled SVR12 of 94.6% (95% CI 92.8–96.4% *I*^*2*^ = 47%; Fig. 5b and c) [20, 24–30]. And the SVR12 of SOF + RBV for 16 weeks is 92.0% (95% CI 81.72–98.74%), which shows lower efficacy with longer treatment duration. Among the 425 patients who received SOF + RBV, 74 (17.4%) had anaemia, 69 (16.2%) had fatigue, 65 (15.3%) had headaches, and 26 types of AEs were reported in 5 studies totally. AEs were experienced by 63.8% of patients, and 13 SAEs occurred among 425 patients.

### Ledipasvir plus Sofosbuvir with a duration of 8 and 12 weeks

The difference was observed when considering patients who received all doses of LDV + SOF for 8 or 12 weeks included from 4 study-arms [29, 31]. Treatment with LDV + SOF for 12 weeks resulted in an SVR12 (96.6%, 95% CI: 92.8-99.2%; Fig. 5d), compared with only 74.1% (95% CI: 54-89%) for a shorter duration of 8 weeks therapy. The most common AEs related to LDV + SOF are nasopharyngitis (14.6%) and headache (11.5%), while pruritus is not common. Specifically, some AEs like upper respiratory tract infection (URTI) (2.5%), gastroenteritis (2.1%), rash (1.9%), diarrhea (1.3%), hyperhidrosis (1.3%), pyrexia (1.3%) and back pain (0.6%) were only recorded in patients who received LDV + SOF.

### Glecaprevir plus Pibrentasvir with a duration of 8 and 12 weeks

For the GLE + PIB regimen, the SVR12 rates were pooled from 4 studies with 693 patients infected with HCV GT2 [30, 32–34]. Comparing the pooled SVR12 with a random effect model between the two duration treatments shows no significant difference in SVR12 rates among the patients treated with 8 weeks (98.0%, 95% CI: 96.6-99.4%; Fig. 5e) and 12 weeks (100%, 95% CI: 99.0-100.0%; Fig. 5f). For safety, 10 kinds of AEs were observed in 455 patients treated with GLE + PIB for 8 or 12 weeks in total. Headache (11.9%), fatigue (8.8%) and nausea (7.9%) are the top three common AEs of patients after receiving all durations of GLE and PIB, while death cases were never observed. The rate of AEs in patients who received treatment with GLE + PIB (60.7%) is lower than the pooled rate (73.1%, 95% CI: 66.6-79.1%).

## Discussion

In this up-to-date, evidence-based systematic review and meta-analysis, we combined SVR12 and AE data from 31 included studies wherever feasible through April 2022. Out of total 3,783 studies identified, we included 13 phase 2 studies and 22 phase 3 or 4 studies. Overall, our meta-analysis confirmed that DAAs therapy is highly effective and safe in the treatment of adult patients with HCV GT2.

The key finding of this study was that both SOF + VEL and GLE + PIB regimens, administered over 12 weeks, achieved the highest efficacy in treating HCV GT2. Additionally, the SOF + VEL regimen demonstrated a high safety profile for patients with GT2, evidenced by the lowest incidence of serious adverse events (1.5%) and comparatively milder side effects. Although both treatments exhibited a relatively high prevalence of adverse events (AEs), the GLE + PIB regimen reported a slightly lower overall AE rate (60.7%) compared to SOF + VEL (68.7%). Among the 58 study-arms, DAAs therapy allows around 80% of GT2 patients reach cure rates ≥ 90%, including those with cirrhosis and who had been treated before. Further analysis revealed an overall pooled SVR12 rate of 94.62% among 2,968 GT2 patients, which aligns with similar results reported for other genotypes [35, 36]. Besides, the therapy against HCV continues evolve. The DAAs regimen like GLE + PIB could shorten the duration into 8 weeks, while reaching 98% SVR12 rate among the GT2 patients. Compared to the 12-week duration of GLE + PIB, the rate of any adverse event in the 8-week group decreased from 65.0 to 56.6%. Shortening the DAA treatment duration for HCV patients can reduce the rate of side effects, there is also research demonstrating that shortened DAA treatment strategies are cost-effective for GT1 patients [37]. There was no observed difference in efficacy among subgroups based on previous treatment history or disease status. These findings align with results from a previous study on other genotypes, in which all patients with GT2 or GT3 were treated with sofosbuvir 400 mg and Velpatasvir 100 mg for 12 weeks and achieved SVR12 [38]. In clinical settings, the selection of a treatment regimen is influenced by various factors, including the accessibility and expense of DAAs, the patient’s tolerance, the potential for adverse reactions or drug-drug interactions, and the occurrence of resistance-associated substitutions (RAS).

However, achieving SVR12 is not the only goal of HCV treatment. While it is an important immediate goal, the ultimate goal is to reduce liver-related mortality and the incidence of hepatocellular carcinoma (HCC) resulting from chronic HCV infection [39]. Among the leading factors of HCC, chronic HCV infection is the primary cause of HCC in Australia [40]. More clinical benefits have been proven with the advent of DAA therapy, which could decrease the recurrence rate of HCC and improve survival [41]. HCV infection is not limited to liver-related symptoms and can also lead to extrahepatic manifestations (EHMs). These manifestations may include mixed cryoglobulinemia, non-Hodgkin lymphomas (NHL), cardiovascular disease, insulin resistance, type 2 diabetes, neurological and psychiatric diseases, and other rheumatic diseases [42]. Studies have shown that DAA treatment can improve or even resolve some EHMs associated with HCV infection [43–46].

In terms of safety, our analysis showed that fatigue (14.0%) was the most common side effect in GT2 patients, followed by headache (13.1%), and the pooled SAE rate was 1.5% (95% CI, 0.8-2.1%). These results confirm that DAAs are safe and well-tolerated. However, some SAEs reported in studies were considered irrelevant to the treatment itself, and therefore, they will not affect the safety evaluation of the DAAs regimen. The most frequent AEs, which included fatigue, headache, pruritus, and nasopharyngitis, were similar across regimens and occurred more frequently in regimens containing RBV. Only the sofosbuvir plus ledipasvir regimen had data for GT2 specifically, with nasopharyngitis (14.6%) as the most common adverse event. Even though the overall AE rates were higher than 50% and may reach 73.1%, the severity was considered mild to moderate, suggesting that treatment interruption or dose adjustment is not required. In assessing the safety profiles of SOF + VEL and GLE + PIB for treating Hepatitis C Virus genotype 2, SOF + VEL showed a 68.7% AE rate over 12 weeks with no treatment-related deaths, while GLE + PIB reported a slightly lower AE rate of 60.7% with no deaths across both 8 and 12-week durations. Despite similar efficacy, GLE + PIB’s marginally better safety profile, characterized by fewer AEs and no severe events, suggests it might be the preferable option, contingent on patient-specific considerations.

Our research has several strengths. It is the first systematic review and meta-analysis involving HCV GT2 patients treated with DAA, which included 35 studies. Moreover, it has a comprehensive subgroup analysis, including different regimens, treatment duration, the presence of cirrhosis, and treatment history. These data also seem to substantiate the suggestion that INF-free DAAs treatment is preferable for patients with any HCV genotypes [47].

There are also limitations to this study. Firstly, two-thirds of the included studies were not randomized controlled trials and were considered to have a low-moderate risk of bias. The single-arm study design has gradually replaced RCTs and becomes the mainstay of DAA therapy clinical trials, resulting in the infeasibility of directly comparing the major agents in DAA combinations. Secondly, the population of GT2 is relatively small, especially when compared to genotypes 1 and 3. Importantly, few studies have categorized patients based on specific genotypes or reported data according to patients’ characteristics, such as genotypes, treatment history, and cirrhosis status. Due to the absence of detailed data, we were unable to conduct further subgroup analyses to assess the efficacy and safety of DAA-based therapies across varying conditions (i.e., treatment history and cirrhosis status). Consequently, it was not feasible to aggregate the sustained virologic response at 12 weeks (SVR12) and the adverse events/serious adverse events (AE/SAE) data for all DAA regimen types. Moreover, only eight of the thirty-five eligible studies provided specific safety data for genotype 2, leading to potential incompleteness and bias in our findings.

## Conclusions

Our systematic review and meta-analysis indicate that DAAs are effective in adults infected with chronic HCV GT2. The efficacy of the DAAs regimen in HCV GT2 patients is independent of patients’ previous treatment history and disease status. Among the various regimens evaluated, the combination of Sofosbuvir plus Velpatasvir for 12 weeks, as well as Glecaprevir plus Pibrentasvir for the same duration, were identified as the most effective and comparatively safe in managing chronic HCV GT2 infection. Both regimens achieved a sustained virological response 12 weeks post-treatment (SVR12) of 100% (95% CI 99–100%).

## Electronic supplementary material

Below is the link to the electronic supplementary material.

Supplementary Material 1

## Data Availability

The data presented in this study are available on request from the corresponding author.
