# Supplementary material for: Efficacy and safety of direct-acting antiviral regimen for patients with hepatitis C virus genotype 2: a systematic review and meta-analysis
Source: BMC Gastroenterol. 2024 Sep 30;24:331. doi: 10.1186/s12876-024-03414-5 (PMC11440749; doi:10.1186/s12876-024-03414-5)

Supplementary Material Appendix

Title: Efficacy and safety of direct-acting antiviral regimen for patients with hepatitis C virus genotype 2: A systematic review and meta-analysis

List of authors:
Pek Kei Lei [1#], Zicheng Liu [1#], Carolina Oi Lam Ung [1,2,3], Hao Hu [1,2,3]*

[1]State Key Laboratory of Quality Research in Chinese Medicine, Institute of Chinese Medical Sciences, University of Macau, Macao SAR, China

[2] Centre for Pharmaceutical Regulatory Sciences, University of Macau, Macao SAR, China

[3] Department of Public Health and Medicinal Administration, Faculty of Health Sciences, University of Macau, Macao SAR, China

[#]co-1st authorship

*Correspondence: haohu@um.edu.mo

Table S1: Full search strategy

	**PubMed**	**21-4-2022**
#1	Search:<br>(((randomized controlled trial[pt] OR controlled clinical trial[pt] OR randomized[tiab] OR placebo[tiab] OR drug therapy[sh] OR randomly[tiab] OR trial[tiab] OR groups[tiab] OR real world OR real life OR single-arm[tiab] or open-label[tiab])) OR (pragmatic trial[tiab])) OR (pragmatic trial)) NOT (animals[mh] NOT humans[mh])<br>Sort by: Relevance	4,796,506
#2	Search:<br>((Hepatitis C, Chronic[Mesh]) OR (Hepatitis C[tiab])) OR (HCV)<br>Sort by: Relevance	96,576
#3	Search:<br>((((((HCV-2) OR ((((G2[tiab] OR GT2[tiab] OR Genotype 2*[tiab] OR genotypes 2*[tiab] OR Genotype 1/2*[tiab] OR Genotypes 1/2*[tiab] OR Genotype 2/3*[tiab] OR Genotypes 2/3*[tiab]))) AND (("Hepatitis C, Chronic"[Mesh] OR Hepatitis c[tiab] OR HCV[tiab]))))))))<br>Sort by: Relevance	3,278
#4	Search:<br>((((((((HCV-2) OR ((((G2[tiab] OR GT2[tiab] OR Genotype 2*[tiab] OR genotypes 2*[tiab] OR Genotype 1/2*[tiab] OR Genotypes 1/2*[tiab] OR Genotype 2/3*[tiab] OR Genotypes 2/3*[tiab]))) AND (("Hepatitis C, Chronic"[Mesh] OR Hepatitis c[tiab] OR HCV[tiab])))))))))) AND (((((randomized controlled trial[pt] OR controlled clinical trial[pt] OR randomized[tiab] OR placebo[tiab] OR drug therapy[sh] OR randomly[tiab] OR trial[tiab] OR groups[tiab] OR real world OR real life OR single-arm[tiab] or open-label[tiab])) OR (pragmatic trial[tiab])) OR (pragmatic trial)) NOT (animals[mh] NOT humans[mh])))<br>Sort by: Relevance	1,882
#5	Search:<br>((((((((((HCV-2) OR ((((G2[tiab] OR GT2[tiab] OR Genotype 2*[tiab] OR genotypes 2*[tiab] OR Genotype 1/2*[tiab] OR Genotypes 1/2*[tiab] OR Genotype 2/3*[tiab] OR Genotypes 2/3*[tiab]))) AND (("Hepatitis C, Chronic"[Mesh] OR Hepatitis c[tiab] OR HCV[tiab])))))))))) AND (((((randomized controlled trial[pt] OR controlled clinical trial[pt] OR randomized[tiab] OR placebo[tiab] OR drug therapy[sh] OR randomly[tiab] OR trial[tiab] OR groups[tiab] OR real world OR real life OR single-arm[tiab] or open-label[tiab])) OR (pragmatic trial[tiab])) OR (pragmatic trial)) NOT (animals[mh] NOT humans[mh]))))) NOT ((((((case study) OR (case report[tiab])) OR (lettter)) OR (Interferon[tiab])) OR (IFN[tiab])))<br>Sort by:Relevance	685

	Embase	21-4-2022
1	'chronic hepatitis'/exp	54,940
2	'hepatitis c':ab,kw,ti	123,787
3	'hep* c':ab,kw,ti	124,774
4	'hepatitis c virus':ab,kw,ti	77,110
5	'hcv':ab,kw,ti	106,218
6	#1 OR #2 OR #3 OR #4 OR #5	187,152
7	'genotypes 2'	1,168
8	'genotype 2'	5,155
9	'genotype 2/3'	575
10	'genotypes 2/3'	248
11	'genotypes 1/2'	457
12	'genotype 1/2'	367
13	'gt2'	1,027
14	'g2'	118,493
15	'g?2'	40,829
16	#7 OR #8 OR #9 OR #10 OR #11 OR #12 OR #13 OR #14 OR #15	163,908
17	'hcv-2/3'	84
18	'hcv 2/3'	84
19	'hcv2/3'	7
20	'hcv 2'	667
21	'hcv-2'	670
22	'hcv2'	266
23	'hcv-1/2'	34
24	'hcv 1/2'	34
25	'hcv1/2'	3
26	'hepatitis c virus genotype 2'/exp	1,635
27	#17 OR #18 OR #19 OR #20 OR #21 OR #22 OR #23 OR #24 OR #25 OR #26	2,163
28	#6 AND #16	6,835
29	#27 OR #28	7,464
30	'clinical trial'	1,718,779
31	'randomized controlled trial'	946,915
32	'randomization'	126,036
33	'single blind procedure'	46,065
34	'double blind procedure'	194,940
35	'crossover procedure'	70,100
36	'randomi?ed controlled trial$':ab,ti,tn	282,336
37	'randomi?ed controlled trial$'	998,429
38	'rct':ab,ti,tn	46,894
39	placebo	501,672

40	'random allocation':ab,ti,tn	2,351
41	'randomly allocated':ab,ti,tn	41,101
42	'allocated randomly':ab,ti,tn	2,788
43	'single blind':ab,ti,tn	20,610
44	'double blind':ab,ti,tn	212,247
45	'triple blind':ab,ti,tn	1,195
46	'placebo$':ab,ti,tn	341,792
47	'prospective study'	824,589
48	'pragmatic':ab,ti,tn	27,701
49	'pragmatic clinical trial':ab,ti,tn	431
50	'single-arm trial':ab,ti,tn	1,499
51	'single-arm':ab,ti,tn	22,654
52	'open-label':ab,ti,tn	96,202
53	'real life':ab,ti,tn	41,322
54	'real world':ab,ti,tn	102,892
55	#30 OR #31 OR #32 OR #33 OR #34 OR #35 OR #36 OR #37 OR #38 OR #39 OR #40 OR #41 OR #42 OR #43 OR #44 OR #45 OR #46 OR #47 OR #48 OR #49 OR #50 OR #51 OR #52 OR #53 OR #54	3,198,786
56	'case study'	178,393
57	'case report':ab,ti,tn	497,607
58	letter	1,311,405
59	'interferon':ab,ti,tn	206,054
60	'ifn':ab,ti,tn	173,876
61	#56 OR #57 OR #58 OR #59 OR #60	2,261,983
62	#55 NOT #61	3,064,892
63	#29 AND #62	1,137

	Web of Science	21-4-2022
#1	((((((TS=(genotypes NEAR/2 3)) OR TS=(genotype NEAR/2 2)) OR TS=(G2)) OR TS=(GT2)) OR TS=(GT NEAR/2 2)) OR TS=(G NEAR/2 2)) OR TS=(HCV NEAR/2 2)	424,124
#2	((((((((((((((TS=(clinical trial)) OR TS=(randomi?ed clinical trial)) OR TS=(randomi?ed controlled trial)) OR TS=(clinical study)) OR TS=(clinical research)) OR TS=(controlled clinical trial)) OR TS=(placebo)) OR TS=(random*)) OR TS=(randomi?ed)) OR TS=(trial)) OR TS=(pragmatic trial)) OR TS=(pragmatic clinical trial)) OR TS=(real life)) OR TS=(real world)) OR TS=(multicent* study)) OR TS=(RCT)	5,251,118
#3	(((((TS=(hepatitis c)) OR TS=(chronic hepatitis c)) OR TS=(HCV)) OR TS=(hepatitis c virus)) OR TS=(Hep* C)) OR TS=(hepatitis NEAR C)	383,962
#4	((((TS=(case study)) OR TS=(case report)) OR TS=(letter)) OR TS=(Interferon)) OR TS=(IFN)	3,775,234
#5	#3 AND #2 AND #1	3,624
#6	#5 NOT #4	1,871

	Cochrane Library	21-4-2022
#1	MeSH descriptor: [Hepatitis C, Chronic] explode all trees	1996
#2	(Genotype 2):ti,ab,kw OR (G2):ti,ab,kw OR (HCV-2):ti,ab,kw OR (GT2):ti,ab,kw OR (Genotypes 2):ti,ab,kw	13,042
#3	("randomized controlled trial"):pt OR ("controlled clinical trial"):pt OR (single-arm):ti,ab,kw OR (open-label):ti,ab,kw OR (real world):ti,ab,kw	684,710
#4	#1 AND #2 AND #3	744
#5	("interferon"):ti,ab,kw OR (IFN):ti,ab,kw	18,605
#6	#4 NOT #5	103

	China National Knowledge Infrastructure, CNKI	21-4-2022
#1	丙型肝炎，主题检索	17,630
#2	2 型，主题检索	138,721
#3	直接抗病毒, 全文检索	9,534
#4	#1 AND #2 AND #3	79

	Clinicaltrials.gov	21-4-2022
#1	Condition or disease: Hepatitis C, Chronic	
#2	Other terms: Genotype 2	
#3	Study type: Interventional Studies (Clinical Trials)	
#4	#1 AND #2 AND #3	610

Table S2: Risk of bias assessment of 26 non-randomized clinical studies included in the systematic review and meta-analysis by using ROBINS-I

	At intervention	Post-intervention			
**Study Name**	**Bias in measurement classification of interventions**	**Bias due to deviations from intended interventions**	**Bias due to missing data**	**Bias in measurement of outcomes**	**Bias in selection of the reported result**
Lawtiz et al., 2015	Low risk (documented HCV infection and treatment)	Moderate risk (2 lost to follow-up and 2 discontinued treatment among 20 patients)	Moderate risk (results were reported for 17 out of 20 patients)	Low risk (laboratory parameter)	Low risk (SVR12 reported for all patients)
Lawitz et al., 2013	Low risk (documented HCV infection and treatment)	Low risk (no deviations in the intervention)	Low risk(all patients completed treatment and follow-up and were included in the analysis)	Low risk (laboratory parameter)	Low risk (SVR12 reported for all patients)
Omata et al., 2014	Low risk (documented HCV infection and treatment)	Low risk (no deviations in the intervention)	Low risk(No patient in the study discontinued treatment prematurely)	Low risk (laboratory parameter)	Low risk (SVR12 reported for all patients)
Brown et al., 2017	Low risk (documented HCV infection and treatment)	Moderate risk (2 out of 56 patients lost to follow-up)	Low risk(No patient in the study discontinued treatment prematurely)	Low risk (laboratory parameter)	Low risk (SVR12 reported for all patients)
Everson et al., 2015	Low risk (documented HCV infection and treatment)	Low risk (no deviations in the intervention)	Low risk(all patients completed treatment and follow-up and were included in the analysis)	Low risk (laboratory parameter)	Low risk (SVR12 reported for all patients)
Foster et al., 2015	Low risk (documented HCV infection and treatment)	Moderate risk(2 were lost to follow-up and 1 discontinued treatment among 132 patients)	Moderate risk (results were reported for 263 out of 266 patients)	Low risk (laboratory parameter)	Low risk (SVR12 reported for all patients)
Foster et al., 2015	Low risk (documented HCV infection and treatment)	Low risk (no deviations in the intervention)	Low risk(No patient in the study discontinued treatment prematurely)	Low risk (laboratory parameter)	Low risk (SVR12 reported for all patients)
Asselah et al., 2018 (SURVEYOR-II)	Low risk (documented HCV infection and treatment)	Moderate risk (1 out of 145 patients lost to follow-up)	Moderate risk (results were reported for 144 out of 145 patients)	Low risk (laboratory parameter)	Low risk (SVR12 reported for all patients)
Gane et al., 2016	Low risk (documented HCV infection and treatment)	Moderate risk (1 out of 33 patients lost to follow-up)	Moderate risk (results were reported for 32 out of 33 patients)	Low risk (laboratory parameter)	Low risk (SVR12 reported for all patients)
Lawitz et al., 2017	Low risk (documented HCV infection and treatment)	Moderate risk (1 lost to follow-up and 1 patients discontinued treatment among 151 patients)	Moderate risk (results were reported for 149 out of 151 patients)	Low risk (laboratory parameter)	Low risk (SVR12 reported for all patients)
Toyoda et al., 2018	Low risk (documented HCV infection and treatment)	Moderate risk (1 lost to follow-up and 3 patients discontinued treatment among 154 patients)	Low risk (authors reported results using ITT analysis)	Low risk (laboratory parameter)	Low risk (SVR12 reported for all patients)

Gane et al., 2017	Low risk (documented HCV infection and treatment)	Low risk (no deviations in the intervention)	Low risk(No patient in the study discontinued treatment prematurely)	Low risk (laboratory parameter)	Low risk (SVR12 reported for all patients)
Ho et al., 2017	Low risk (documented HCV infection and treatment)	Moderate risk (1 lost to follow-up and 7 discontinued treatment among 66 patients)	Moderate risk (results were reported for 58 out of 66 patients)	Low risk (laboratory parameter)	Low risk (SVR12 reported for all patients)
Jacobson et al., 2017	Low risk (documented HCV infection and treatment)	Low risk (no deviations in the intervention)	Low risk(No patient in the study discontinued treatment prematurely)	Low risk (laboratory parameter)	Low risk (SVR12 reported for all patients)
Lawitz et al., 2019	Low risk (documented HCV infection and treatment)	Moderate risk (2 lost to follow-up and 1 withdrawal among 47 patients)	Moderate risk (results were reported for 44 out of 47 patients)	Low risk (laboratory parameter)	Low risk (SVR12 reported for all patients)
Lawitz et al., 2019	Low risk (documented HCV infection and treatment)	Moderate risk (1 out of 29 patients discontinued treatment)	Moderate risk (results were reported for 28 out of 29 patients)	Low risk (laboratory parameter)	Low risk (SVR12 reported for all patients)
Wei et al., 2018	Low risk (documented HCV infection and treatment)	Low risk (no deviations in the intervention)	Low risk(No patient in the study discontinued treatment prematurely)	Low risk (laboratory parameter)	Low risk (SVR12 reported for all patients)
Wei et al.,2020 (VOYAGE-2)	Low risk (documented HCV infection and treatment)	Moderate risk (1 out of 139 patients missed SVR12 data)	Moderate risk (1 out of 139 patients missed SVR12 data)	Low risk (laboratory parameter)	Low risk (SVR12 reported for all patients)
Asahina et al., 2018	Low risk (documented HCV infection and treatment)	Moderate risk (2 discontinued treatment and 1 lost to follow-up among 239 patients)	Moderate risk (results were reported for 236 out of 239 patients)	Low risk (laboratory parameter)	Low risk (SVR12 reported for all patients)
Izumi et al., 2018	Low risk (documented HCV infection and treatment)	Low risk (no deviations in the intervention)	Low risk(No patient in the study discontinued treatment prematurely)	Low risk (laboratory parameter)	Low risk (SVR12 reported for all patients)
Rao et al., 2019	Low risk (documented HCV infection and treatment)	Low risk (no deviations in the intervention)	Low risk(No patient in the study discontinued treatment prematurely)	Low risk (laboratory parameter)	Low risk (SVR12 reported for all patients)
Shafran et al., 2017	Low risk (documented HCV infection and treatment)	Low risk (no deviations in the intervention)	Low risk (authors reported results using ITT analysis)	Low risk (laboratory parameter)	Low risk (SVR12 reported for all patients)
Brown et al., 2020	Low risk (documented HCV infection and treatment)	Low risk (no deviations in the intervention)	Low risk (authors reported results using ITT analysis)	Low risk (laboratory parameter)	Low risk (SVR12 reported for all patients)

Asselah et al.,2019	Low risk (documented HCV infection and treatment)	Low risk (no deviations in the intervention)	Low risk(No patient in the study discontinued treatment prematurely)	Low risk (laboratory parameter)	Low risk (SVR12 reported for all patients)
Gao et al.,2020	Low risk (documented HCV infection and treatment)	Low risk (no deviations in the intervention)	Low risk(No patient in the study discontinued treatment prematurely)	Low risk (laboratory parameter)	Low risk (SVR12 reported for all patients)
Hua et al., 2022	Low risk (documented HCV infection and treatment)	Low risk (no deviations in the intervention)	Low risk(No patient in the study discontinued treatment prematurely)	Low risk (laboratory parameter)	Low risk (SVR12 reported for all patients)

Table S3: Risk of bias assessment of 9 randomized controlled clinical studies included in the systematic review and meta-analysis by using ROB 2 tool

Study	Random sequence generation (selection bias)	Allocation concealment (selection bias)	Blinding of participants and personnel (performance bias)	Blinding of outcome assessment (detection bias)	Incomplete outcome data (attrition bias)	Selective reporting (reporting bias)	Other bias	Overall
Jacobson et al., 2013 (POSITRON)	Low	Low	Low	Low	Low	Low	High	Low
Jacobson et al., 2013 (FUSION)	Low	Low	Low	Low	Low	Low	High	Low
Zeuzem et al., 2014 (VALENCE)	High	High	High	Low	Low	Low	High	High
Foster et al., 2016 (PRINCIPAL)	Low	Low	Low	Unclear	Low	Low	High	Low
Dore et al., 2015 (AI444-031)	Low	Low	Unclear	Low	Low	Low	High	Low
Feld et al., 2015 (ASTRAL-1)	Low	Low	Low	Low	Low	Low	High	Low
Asselah et al., 2018 (ENDURANCE-2)	Low	Low	Low	Low	Low	Low	High	Low
Toyoda et al., 2018 (CERTAIN-2)	High	High	High	Unclear	Low	Low	High	Low
Wei et al., 2020 (VOYAGE-1)	Low	Low	Low	Low	Low	Low	High	Low

Table S4: Characteristics of 58 study-arms included in the systematic review and meta-analysis

Arm	Study	Intervention	Treatment-naïve		Treatment-experienced		No Cirrhosis		Cirrhosis		Overall	
1	2.Lawtiz et al., 2015	OBV+PTV+RTN+RBV-12	8	10			8	10			8	10
2	2.Lawtiz et al., 2015	OBV+PTV+RTN-12	6	10			6	10			6	10
3	6.Jacobson et al., 2013	SOF+RBV-12	101	109			85	92	16	17	101	109
4	6.Jacobson et al., 2013	SOF+RBV-12			31	36	25	26	6	10	31	36
5	6.Jacobson et al., 2013	SOF+RBV-16			30	32	23	23	7	9	30	32
6	7.Lawitz et al., 2013	SOF+RBV-12	68	70				20		50	68	70
7	9.Zeuzem et al., 2014	SOF+RBV-12	31	32	37	41	59	63	9	11	68	73
8	23.Foster et al., 2016	Lambda+RBV+DCV-12	165	184			154	172	11	12	165	184
9	24.Omata et al., 2014	SOF+RBV-12	88	90	60	63	132	136	16	17	148	153
10	25.Brown et al., 2017	EBR+GZR + RBV-12	24	30			24	30			24	30
11	25.Brown et al., 2017	GZR + RBV-12	19	26			19	26			19	26
12	34.Dore et al., 2015	DCV+PEG+RBV-12	21	24							21	24
13	34.Dore et al., 2015	DCV+PEG+RBV-16	19	23							19	23
14	35.Everson et al., 2015	SOF+VEL25-8	20	26			20	26			20	26
15	35.Everson et al., 2015	SOF+VEL25+RBV-8	22	25			22	25			22	25
16	35.Everson et al., 2015	SOF+VEL100-8	23	26			23	26			23	26
17	35.Everson et al., 2015	SOF+VEL100+RBV-8	23	26			23	26			23	26
18	37.Feld et al., 2015	SOF+VEL-12			104	104	93	93	10	10	104	104
19	39.Foster et al., 2015	SOF+VEL-12		115		19		115		19	133	134
20	39.Foster et al., 2015	SOF+RBV-12		112		20		113		19	124	132
21	40.Foster et al., 2015	SOF+RBV-16									13	15
22	40.Foster et al., 2015	SOF+RBV-24									17	17
23	40.Foster et al., 2015	SOF+RBV+PEG-12									15	16
24	47.Asselah et al., 2018	GLE+PIB-8		127		18	142	145			142	145
25	47.Asselah et al., 2018	GLE+PIB-12		141		61					201	202
26	51.Gane et al., 2016	SOF+VEL+VOX-6					4	6			4	6
27	51.Gane et al., 2016	SOF+VEL+VOX-8			6	6			6	6	6	6
28	51.Gane et al., 2016	SOF+VEL+VOX-12					13	13	8	8	21	21

29	55.Lawitz et al., 2017	GZR+RZR+UPR-8	**29**	**32**			**29**	**32**			**29**	**32**
30	55.Lawitz et al., 2017	GZR+RZR+UPR+RBV-8	**25**	**31**			**25**	**31**			**25**	**31**
31	55.Lawitz et al., 2017	GZR+RZR+UPR-12	**45**	**46**			**30**	**31**	**15**	**15**	**45**	**46**
32	55.Lawitz et al., 2017	GZR+RZR+UPR+RBV-12	**16**	**16**					**16**	**16**	**16**	**16**
33	55.Lawitz et al., 2017	GZR+RZR+UPR-16	**26**	**26**					**26**	**26**	**26**	**26**
34	59.Toyoda et al., 2018	GLE+PIB-8		**75**		**15**	**88**	**90**			**88**	**90**
35	59.Toyoda et al., 2018	SOF+RBV-12		**38**		**8**	**43**	**46**			**43**	**46**
36	59.Toyoda et al., 2018	GLE+PIB-12		**11**		**7**			**18**	**18**	**18**	**18**
37	64.Gane et al., 2017	LDV+SOF-8	**15**	**20**	**5**	**7**	**20**	**27**			**20**	**27**
38	64.Gane et al., 2017	LDV+SOF-12		**21**		**5**		**24**		**2**	**25**	**26**
39	65.Ho et al., 2017	SOF+RBV-12	**36**	**47**	**16**	**19**			**52**	**66**	**52**	**66**
40	66.Jacboson et al., 2017	SOF+VEL+VOX-8	**61**	**63**							**61**	**63**
41	66.Jacboson et al., 2017	SOF+VEL-12	**53**	**53**							**53**	**53**
42	69.Lawitz et al., 2019	RZR180+UPR-12		**45**		**2**	**32**	**34**	**10**	**10**	**43**	**47**
43	70.Lawitz et al., 2019	RZR60+UPR-12	**26**	**27**	**2**	**2**	**21**	**22**	**7**	**7**	**28**	**29**
44	77.Wei et al., 2020	GLE+PIB-8					**136**	**139**			**136**	**139**
45	77.Wei et al., 2020	GLE+PIB-12							**53**	**53**	**53**	**53**
46	80.Asahina et al., 2018	LDV+SOF-12	**70**	**72**	**32**	**34**	**89**	**92**	**13**	**14**	**102**	**106**
47	80.Asahina et al., 2018	SOF+RBV-12	**74**	**74**	**29**	**34**	**87**	**92**	**16**	**16**	**103**	**108**
48	80.Asahina et al., 2018	LDV+SOF-12	**12**	**13**	**12**	**12**	**21**	**21**	**3**	**4**	**24**	**25**
49	89.Izumi et al., 2018	SOF+VEL+RBV-12			**7**	**10**		**8**		**2**	**7**	**10**
50	89.Izumi et al., 2018	SOF+VEL+RBV-24			**11**	**12**		**9**		**3**	**11**	**12**
51	95.Rao et al., 2019	CLV30+SOF-12	**8**	**8**							**8**	**8**
52	95.Rao et al., 2019	CLV+SOF-12	**18**	**19**							**18**	**19**
53	97.Shafran et al., 2017	OBV+PTV+RTN+SOF+RBV-8	**8**	**8**	**2**	**2**	**9**	**10**			**9**	**10**
54	97.Shafran et al., 2017	OBV+PTV+RTN+SOF+RBV-6		**7**		**2**	**4**	**9**			**4**	**9**
55	101.Brown et al., 2020	GLE+PIB-8	**26**	**26**					**26**	**26**	**26**	**26**
56	102.Asselah et al., 2019	SOF+VEL-12									**20**	**20**
57	126.Gao et al., 2020	CLV+SOF-12	**91**	**95**			**81**	**85**	**10**	**10**	**91**	**95**
58	140.Hua et al., 2022	AFV+DCV-12									**31**	**31**
	**Total**			**2079**		**571**		**1998**		**476**	**2791**	**2968**

Table S5: Full meta-analysis of proportions of selected Adverse Events for subjects aged 0-18 years with chronic hepatitis C virus (HCV) infection and treated with Direct-Acting Antivirals (DAAs)

**Common AEs**	**DAA regimen and No. of event/No. of participants**									**Proportion (95% CI) I^2 statistic (*P* value)**
	OBV+PTV+RTN+SOF+RBV-8 (n=1)	OBV+PTV+RTN+SOF+RBV-6 (n=1)	EBR/GZR+RBV-12 (n=1)	SOF+VEL-12 (n=1)	LDV+SOF-8 (n=1)	LDV+SOF-12 (n=3)	SOF+RBV-12 (n=5)	GLE+PIB-8 (n=2)	GLE+PIB-12 (n=2)	
Fatigue	3/10	6/9	12/30	20/134	5/27	6/157	69/425	17/235	23/220	14.0% [6.4; 21.6]<br>92.8% (p＜0.0001)
Headache	6/10	3/9	6/30	24/134	8/27	18/157	65/425	30/235	24/220	13.1% [9.2; 17.1]<br>76.7% (p＜0.0001)
Nausea	3/10		5/30	14/134	5/27	7/157	31/425	21/235	15/220	5.7% [2.2; 10.8]<br>89.6% (p＜0.0001)
Insomnia	2/10			6/134			22/425			0.2% [0.0; 0.6]<br>52.0% (p＜0.001)
Irritability				4/134			9/425			0.1% [0.0; 0.5]<br>0% (p=0.64)
Pruritus		2/9		6/134			18/425	3/235	4/220	1.3% [0.2; 2.4]<br>53.3% (p＜0.01)
Nasopharyngitis				8/134		23/157	76/425	9/235	2/220	7.4% [2.5; 12.4]<br>89.3% (p＜0.001)
Cough			5/30	4/134			6/425			0.1% [0; 0.5]<br>0% (p=0.45)
Dyspepsia				1/134	2/27		9/425			0.1% [0.0; 0.5]<br>0% (p=0.74)
Rash					2/27	3/157	5/425			0.1% [0; 0.4]<br>0% (p=0.82)
Gastroenteritis					2/27	2/157				0%[0; 0]<br>0% (p=0.48)
Upper respiratory tract infection	4/10					4/157				0%[0; 0]<br>51.5% (p＜0.01)
Back pain					2/27	1/157				0%[0; 0.36]<br>0 (p=0.99)
Diarrhea	3/10	2/9			1/27	2/157	4/425			0.1%[0; 0.4]<br>0% (p=0.58)
Vomiting					1/27	2/157	4/425			0%[0; 0.2]<br>29.7% (p=0.12)

Hyperhidrosis						2/157				0%[0; 0]<br>0% (p=0.91)
Anaemia			5/30				74/425		1/220	5.7% [1.2; 10.3]<br>(p＜0.0001)
Stomatitis						6/157	5/425	1/235		0.14% [0; 0.5]<br>0% (p= 0.74)
Pyrexia						2/157				0% [0; 0]<br>0 (p=0.90)
Blood bilirubin increased							7/425	1/235	2/220	2% [0; 0.8]<br>58.3% (p＜0.01)
Malaise							15/425	5/235	1/220	0.1% [0; 0.5]<br>27.9% (p=0.14)
Hyperuricemia							3/425			0% [0; 0]<br>0 (p=0.77)
Dizziness	1/10	2/9					6/425			0% [0; 0.4]<br>0% (p= 0.85)
Dyspnea							5/425			0% [0; 0.01]<br>0% (p= 0.39)
Depression							4/425			0% [0; 0]<br>0 (p= 0.62)
Musculoskeletal pain							4/425			0% [0; 0]<br>0 (p= 0.62)
**SAE**	1/10	0	1/30	2/134	2/27	4/157	13/425	3/235	3/220	1.5% [0.8; 2.1]<br>0% (p=0.64)
**Death**	0	0	0	2	0	1	0	0	0	0% [0; 0.2]<br>0 (p=0.87)
**Any AE**	10/10<br>(100%)	9/9<br>(100%)	26/30<br>(86.7%)	92/134<br>(68.7%)	22/27<br>(81.5%)	95/157<br>(60.5%)	271/425(<br>63.8%)	133/235<br>(56.6%)	143/220<br>(65.0%)	73.1% [66.6; 79.1]<br>77.4% (p＜0.0001)

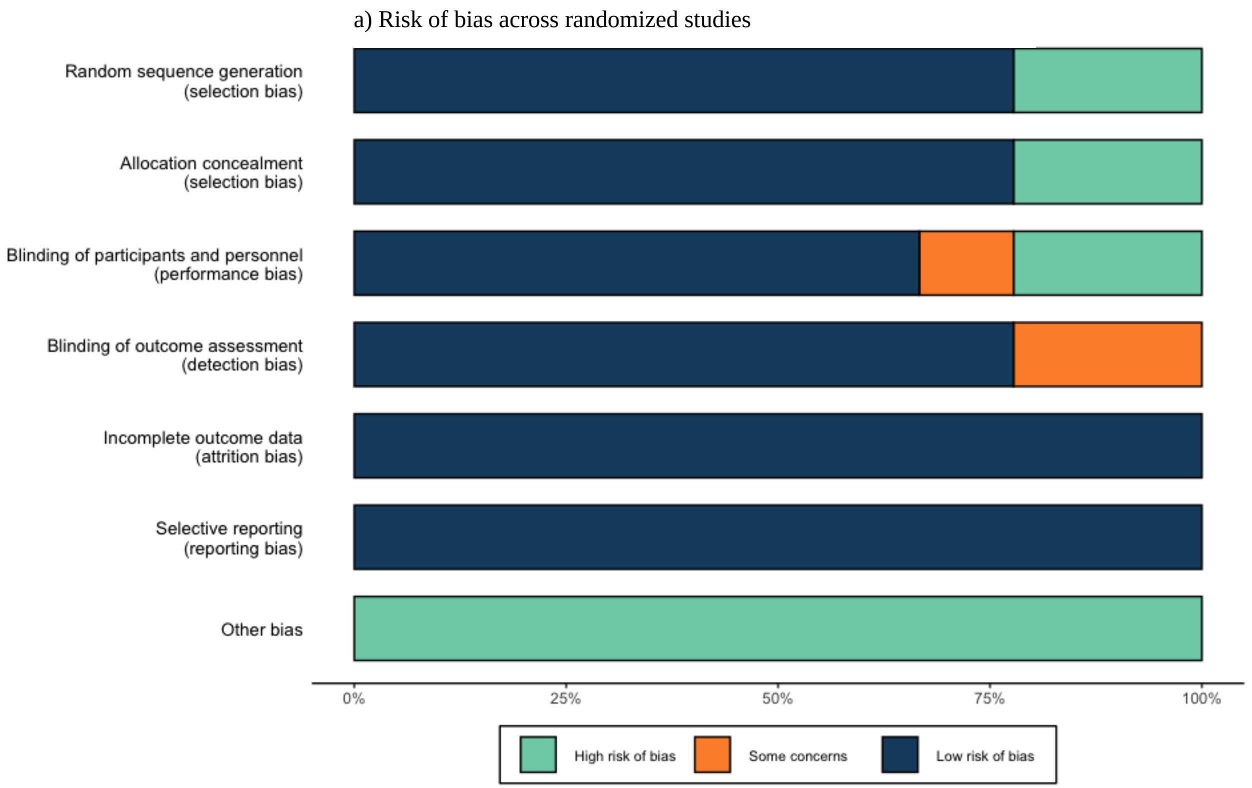

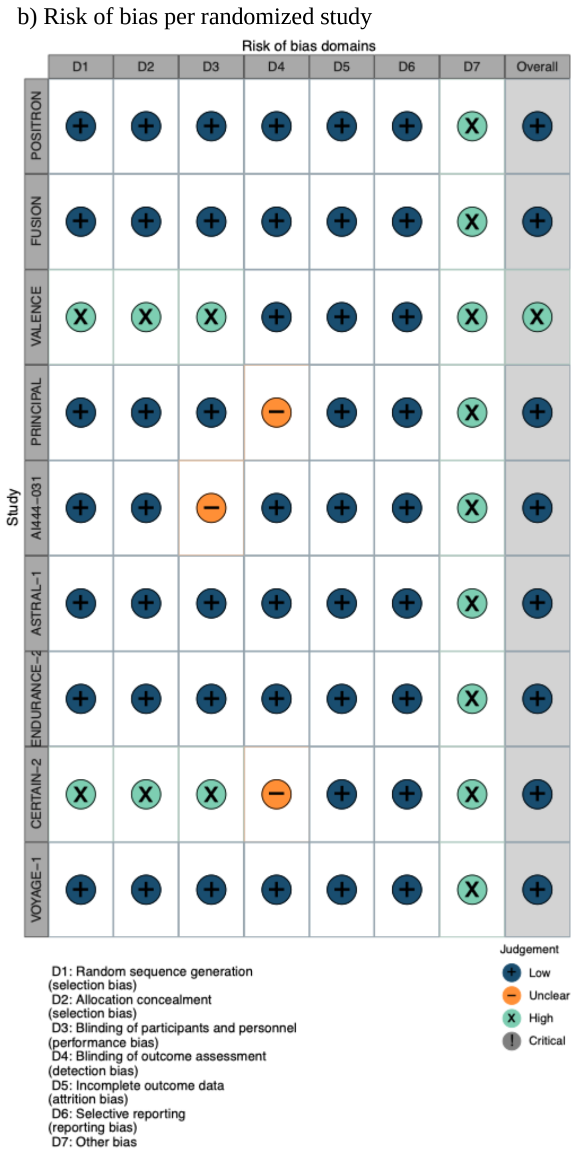

Figure S1: Quality assessment of included studies using the ROB 2. (a) Risk of bias across randomized studies. (b) Risk of bias per randomized study

Figure S2 Forest plot of proportions of HCV GT2 patients reaching SVR12 with DAAs

Study	Events	Total	SVR12(%)	95%–CI	Weight (common)	Weight (random)
Feld et al., 2015	104	104	100.00	[96.52; 100.00]	3.5%	2.3%
Foster et al., 2015	17	17	100.00	[80.49; 100.00]	0.6%	1.4%
Gane et al., 2016	6	6	100.00	[54.07; 100.00]	0.2%	0.7%
Gane et al., 2016	21	21	100.00	[83.89; 100.00]	0.7%	1.5%
Lawitz et al., 2017	16	16	100.00	[79.41; 100.00]	0.6%	1.3%
Lawitz et al., 2017	26	26	100.00	[86.77; 100.00]	0.9%	1.6%
Toyoda et al., 2018	18	18	100.00	[81.47; 100.00]	0.6%	1.4%
Jacboson et al., 2017	53	53	100.00	[93.28; 100.00]	1.8%	2.0%
Wei et al., 2020	53	53	100.00	[93.28; 100.00]	1.8%	2.0%
Rao et al., 2019	8	8	100.00	[63.06; 100.00]	0.3%	0.9%
Brown et al., 2020	26	26	100.00	[86.77; 100.00]	0.9%	1.6%
Asselah et al., 2019	20	20	100.00	[83.16; 100.00]	0.7%	1.5%
Hua et al., 2022	31	31	100.00	[88.78; 100.00]	1.1%	1.7%
Asselah et al., 2018	201	202	99.50	[97.27; 99.99]	6.8%	2.4%
Foster et al., 2015	133	134	99.25	[95.91; 99.98]	4.5%	2.4%
Asselah et al., 2018	142	145	97.93	[94.07; 99.57]	4.9%	2.4%
Wei et al., 2020	136	139	97.84	[93.82; 99.55]	4.7%	2.4%
Lawitz et al., 2017	45	46	97.83	[88.47; 99.94]	1.6%	2.0%
Toyoda et al., 2018	88	90	97.78	[92.20; 99.73]	3.0%	2.2%
Lawitz et al., 2013	68	70	97.14	[90.06; 99.65]	2.4%	2.1%
Jacboson et al., 2017	61	63	96.83	[89.00; 99.61]	2.1%	2.1%
Omata et al., 2014	148	153	96.73	[92.54; 98.93]	5.1%	2.4%
Lawitz et al., 2019	28	29	96.55	[82.24; 99.91]	1.0%	1.7%
Asahina et al., 2018	102	106	96.23	[90.62; 98.96]	3.6%	2.3%
Gane et al., 2017	25	26	96.15	[80.36; 99.90]	0.9%	1.6%
Asahina et al., 2018	24	25	96.00	[79.65; 99.90]	0.9%	1.6%
Gao et al., 2020	91	95	95.79	[89.57; 98.84]	3.2%	2.3%
Asahina et al., 2018	103	108	95.37	[89.53; 98.48]	3.6%	2.3%
Rao et al., 2019	18	19	94.74	[73.97; 99.87]	0.7%	1.4%
Foster et al., 2015	124	132	93.94	[88.41; 97.35]	4.4%	2.4%
Jacobson et al., 2013	30	32	93.75	[79.19; 99.23]	1.1%	1.8%
Foster et al., 2015	15	16	93.75	[69.77; 99.84]	0.6%	1.3%
Toyoda et al., 2018	43	46	93.48	[82.10; 98.63]	1.6%	2.0%
Zeuzem et al., 2014	68	73	93.15	[84.74; 97.74]	2.5%	2.2%
Jacobson et al., 2013	101	109	92.66	[86.05; 96.78]	3.7%	2.3%
Izumi et al., 2018	11	12	91.67	[61.52; 99.79]	0.4%	1.1%
Lawitz et al., 2019	43	47	91.49	[79.62; 97.63]	1.6%	2.0%
Lawitz et al., 2017	29	32	90.62	[74.98; 98.02]	1.1%	1.8%
Shafran et al., 2017	9	10	90.00	[55.50; 99.75]	0.4%	1.0%
Foster et al., 2016	165	184	89.67	[84.34; 93.67]	6.2%	2.4%
Everson et al., 2015	23	26	88.46	[69.85; 97.55]	0.9%	1.6%
Everson et al., 2015	23	26	88.46	[69.85; 97.55]	0.9%	1.6%
Everson et al., 2015	22	25	88.00	[68.78; 97.45]	0.9%	1.6%
Dore et al., 2015	21	24	87.50	[67.64; 97.34]	0.8%	1.6%
Foster et al., 2015	13	15	86.67	[59.54; 98.34]	0.5%	1.3%
Jacobson et al., 2013	31	36	86.11	[70.50; 95.33]	1.2%	1.8%
Dore et al., 2015	19	23	82.61	[61.22; 95.05]	0.8%	1.6%
Lawitz et al., 2017	25	31	80.65	[62.53; 92.55]	1.1%	1.7%
Lawtiz et al., 2015	8	10	80.00	[44.39; 97.48]	0.4%	1.0%
Brown et al., 2017	24	30	80.00	[61.43; 92.29]	1.0%	1.7%
Ho et al., 2017	52	66	78.79	[66.98; 87.89]	2.2%	2.1%
Everson et al., 2015	20	26	76.92	[56.35; 91.03]	0.9%	1.6%
Gane et al., 2017	20	27	74.07	[53.72; 88.89]	0.9%	1.6%
Brown et al., 2017	19	26	73.08	[52.21; 88.43]	0.9%	1.6%
Izumi et al., 2018	7	10	70.00	[34.75; 93.33]	0.4%	1.0%
Gane et al., 2016	4	6	66.67	[22.28; 95.67]	0.2%	0.7%
Lawtiz et al., 2015	6	10	60.00	[26.24; 87.84]	0.4%	1.0%
Shafran et al., 2017	4	9	44.44	[13.70; 78.80]	0.3%	1.0%
**Common effect model**		**2968**	**96.49**	**[95.66; 97.25]**	**100.0%**	**--**
**Random effects model**			**94.62**	**[92.43; 96.51]**	**--**	**100.0%**

Heterogeneity: $I^2 = 72\%$, $\tau^2 = 0.0150$, $p < 0.01$

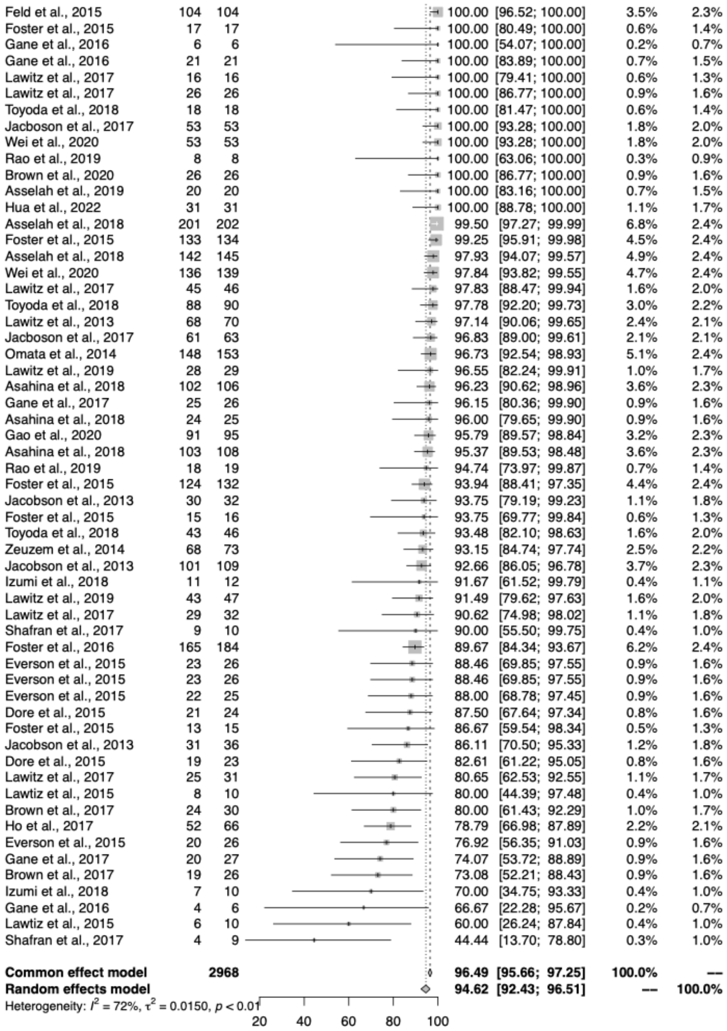

Figure S3: Forest plot of proportions of chronic hepatitis C virus (HCV) genotype 2(GT2) patients reaching Sustained Virologic Response 12 weeks after the end of treatment (SVR12) with Direct-Acting Antivirals (DAAs) over patients receiving all doses of treatment, according to type of treatment. (Only regimens with >1 study are shown)

a) DAA regimen: SOF+VEL+VOX-8

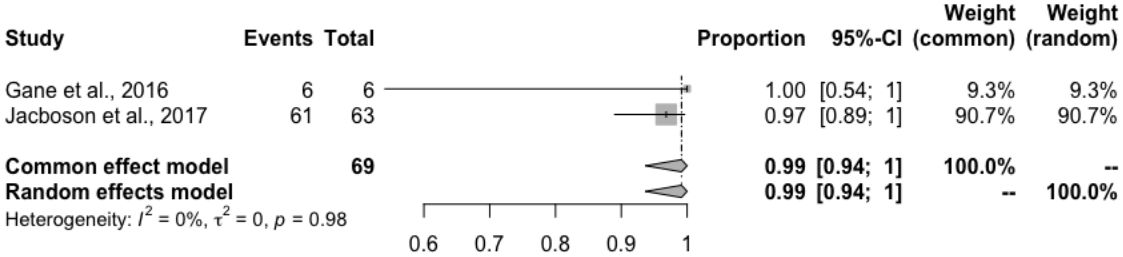

b) DAA regimen: SOF+VEL-12

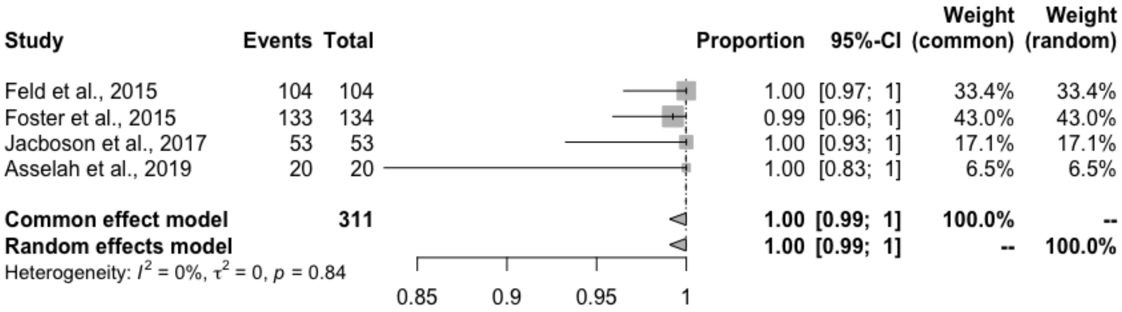

c) DAA regimen: SOF+RBV-16

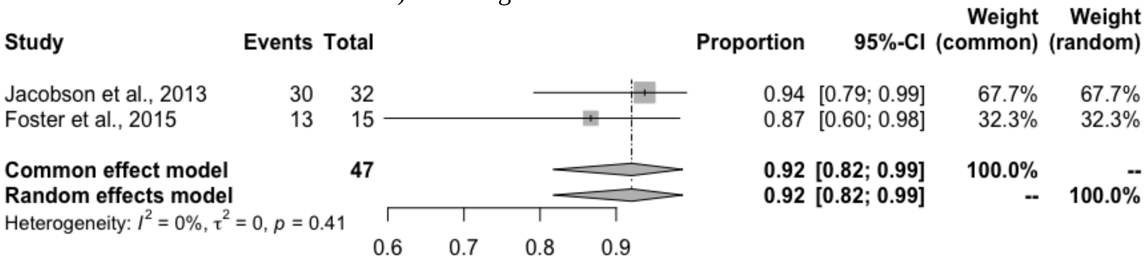

d) DAA regimen:SOF+RBV-12

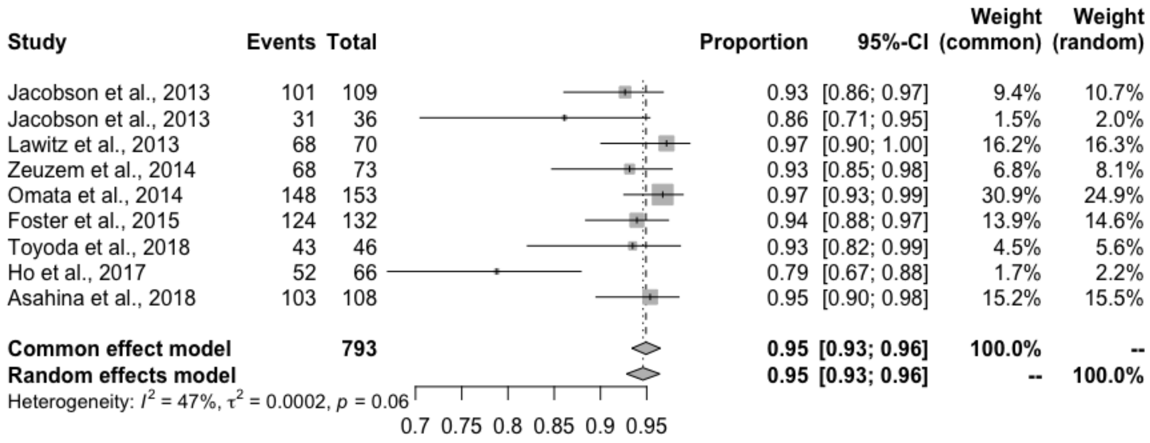

e) DAA regimen: LDV+SOF-12

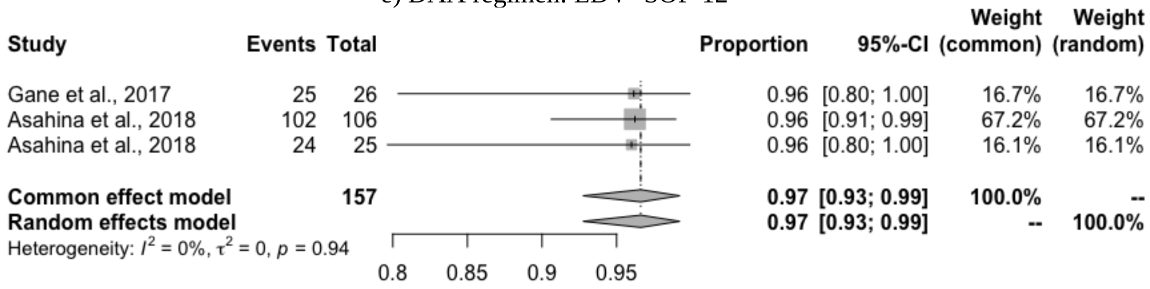

f) DAA regimen: GLE+PIB-12

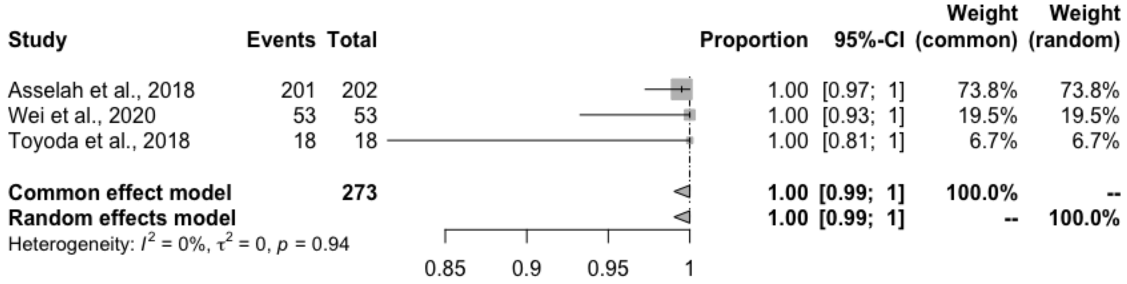

g) DAA regimen: GLE+PIB-8

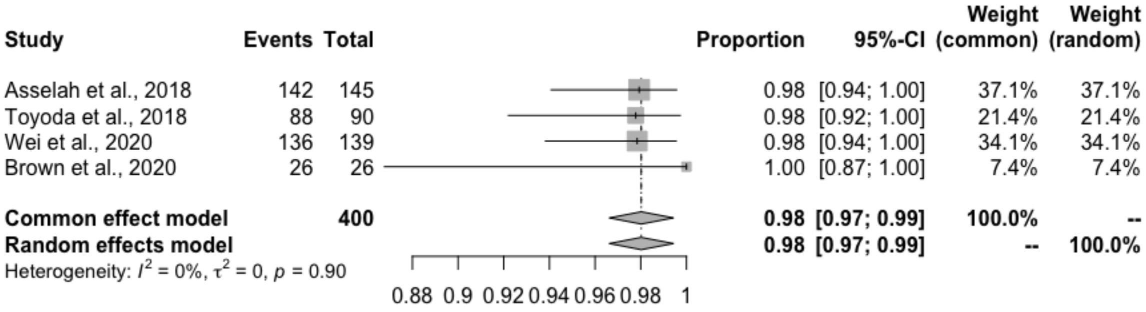

h) DAA regimen 8: CLV+SOF-12

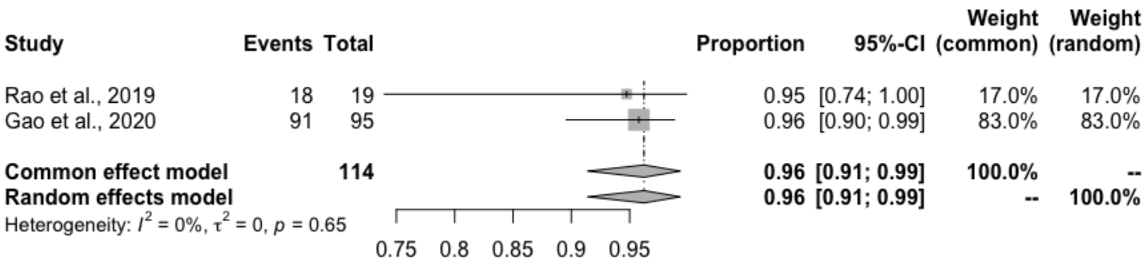

Supplement: Supplementary file 1 — Supplementary Material 1 [file 12876_2024_3414_MOESM1_ESM.pdf]
